# Gut microbiome signature of metabolically healthy obese individuals according to anthropometric, metabolic and inflammatory parameters

**DOI:** 10.1038/s41598-024-53837-z

**Published:** 2024-02-11

**Authors:** Ho-Kyoung Lee, Nam-Eun Kim, Cheol Min Shin, Tae Jung Oh, Hyuk Yoon, Young Soo Park, Nayoung Kim, Sungho Won, Dong Ho Lee

**Affiliations:** 1 Department of Internal Medicine, Seoul National University Bundang Hospital, 82, Gumi-ro 173, Beon-gil, Bundang-gu, Seongnam-si, Gyeonggi-do 13620 South Korea; 2 Institute of Health and Environment, Seoul National University, Seoul, South Korea; 3 Department of Public Health Sciences, Seoul National University, Seoul, South Korea

**Keywords:** Endocrine system and metabolic diseases, Metabolic disorders, Clinical microbiology

## Abstract

In this study, we investigated the characteristics of gut microbiome in the metabolically healthy obese (MHO) patients, and how they correlate with metabolic and inflammatory profiles. A total of 120 obese people without metabolic comorbidities were recruited, and their clinical phenotypes, metabolic and inflammatory parameters were analysed. The faecal microbial markers originating from bacterial cell and extracellular vesicle (EV) were profiled using 16S rDNA sequencing. The total study population could be classified into two distinct enterotypes (enterotype I: Prevotellaceae-predominant, enterotype II: *Akkermansia*/*Bacteroides-*predominant), based on their stool EV-derived microbiome profile. When comparing the metabolic and inflammatory profiles, subjects in enterotype I had higher levels of serum IL-1β [false discovery rate (FDR) q = 0.050] and had a lower level of microbial diversity than enterotype II (Wilcoxon rank-sum test *p* < 0.01). Subjects in enterotype I had relatively higher abundance of Bacteroidetes, Prevotellaceae and *Prevotella-*derived EVs, and lower abundance of Actinobacteria, Firmicutes*,* Proteobacteria*, Akkermansia* and *Bacteroides*-derived EVs (FDR q < 0.05). In conclusion, HMO patients can be categorised into two distinct enterotypes by the faecal EV-derived microbiome profile. The enterotyping may be associated with different metabolic and inflammatory profiles. Further studies are warranted to elucidate the long-term prognostic impact of EV-derived microbiome in the obese population.

## Introduction

Obesity is a chronic condition that brings together multiple metabolic comorbidities including cardiovascular disease, diabetes mellitus, dyslipidemia, and even cancer. Obesity is rapidly increasing worldwide, resulting not only in increased metabolic complications and shortened life expectancy, but also in increased socioeconomic burden^1^. The proportion of the obese population has increased by 27.5% in the adult population and 47.1% in the children population^2^. The World Obesity Atlas 2022 report has estimated that the obese population would increase up to 1 million worldwide by 2030, which accounts for one in every five women and one in every seven men^3^.

Obese populations were previously thought to be a homogenous group with similar metabolic properties and prognosis. Nevertheless, studies have revealed a unique subgroup of obese subjects who are free from various metabolic abnormalities despite high body mass index (BMI), defined as “metabolically healthy obese” (MHO) subjects^4^. Cohort studies on these MHO patients have shown a relatively lower risk of cardiovascular disease, type 2 diabetes, and hypertension as compared to that of metabolically unhealthy obese subjects. Nevertheless, the population still had a higher risk of cardiovascular disease and type 2 diabetes, compared to the metabolically healthy, normal-weight population^1,5–7^. This state is thought to be a dynamic process with a risk of transition to metabolically unhealthy obesity, and studies on clarifying the long-term and optimal management outcomes of this population are underway.

Gut microbes that colonise the human intestinal tract create a mutualistic relationship with the host^8^. Recent studies have revealed that the human gut microbiome greatly influences the response to dietary intake and contributes to the development of various metabolic diseases^9^. Obesity is characterised by the dysbiosis of the gut microbiome^10,11^. The overall diversity of gut microbiota has been reported to decrease with increasing body mass index^12,13^. Moreover, studies on obese populations have also suggested the alterations in the ratio of Firmicutes and Bacteroidetes in the gut microbiome^13–16^. Nevertheless, the reported changes in the Firmicutes and Bacteroidetes ratio are inconsistent across studies, and it remains unclear which specific gut microbial profile is directly related to the development of obesity.

It is suggested that the gut microbial profile in MHO subjects might differ from that of non-obese or metabolically unhealthy subjects. As human gut microbes are well known to interact with host metabolism and inflammation, the microbial composition of the human gut microbiota in MHO subjects might show a distinctive profile compared to that that of metabolically unhealthy subjects. Xiazhong et al*.* have previously reported changes in microbial diversity and beta-diversity in MHO subjects^17^. However, there is insufficient evidence regarding the specific taxonomic changes in MHO subjects, and how they correlate with different metabolic parameters.

Recent studies on the gut microbiome have also revealed that extracellular vesicles (EV) secreted by Gram-negative and Gram-positive bacteria play an important role in the interaction between the host and the human microbiome. These organelles carry a wide variety of bacterial-origin bioactive materials including proteins, lipids, polysaccharides and RNAs, and are believed to influence bacterial and host cell function in an autocrine and paracrine manner, and thereby influence the host metabolism and immune response^18–20^.

Bacteria-derived EVs have been found to induced different metabolic dysfunctions, and thereby leading to the development of diabetes or obesity^21^. These EVs modulated intestinal inflammation and barrier integrity in several in vivo studies^22^. Ashrafian et al. observed that EVs derived from *Akkermansia muciniphila* enhanced intestinal barrier function and reduced the in host inflammatory response^23^. Consistent with this study, Clarithanay Chelakkot et al. also reported that *A. muciniphila*-derived EVs directly enhanced gut permeability and thereby enhanced metabolic functions in high-fat diet-fed mice^24^.

Moreover, the gut microbiome also modulates host immune function by interacting with mucosal-associated immune cells. *Bacteroides fragilis* EVs induced immune tolerance by activating dendritic cells in a colitis model as per a study by Shen et al.^25^. Furthermore, a study by Choi et al. revealed there were changes in bacteria-derived EVs in high-fat diet-fed mice, that were more dramatic as compared to the gut microbe composition^26^.

On the basis of these previous studies, it is postulated that the EV derived microbiome in the stool of obese subjects could have a distinct microbial profile depending on their metabolic and inflammatory status. Through this study, we aimed to observe the characteristics of the bacterial EV-derived microbiome in obese patients with no other metabolic complications. Furthermore, we aimed to reveal how the gut microbial profile correlates with different metabolic and inflammatory features.

## Results

### Baseline characteristics of healthy obese subjects

The clinical characteristics and blood samples of the 120 study subjects were collected for analysis (Table 1). A total of 21 men and 99 women participated in this study. The mean age of the participants was 44 years old, and 7 out of the 120 subjects were smokers. The mean body mass index (BMI), and the median waist circumference (WC), and waist-to-hip ratio (WHR) were measured to be 27.8 kg/m^2^, 95.1 cm and 0.92, respectively.

**Table 1:** Baseline characteristics (N = 120).

Variables	Patient characteristics (mean standard ± deviation)	Reference value
Male sex, n (%)	21 (17.5%)	–
Age (years)	44.0 ± 8.9	–
Smoker, n (%)	7 (5.8%)	–
Consumes alcohol^††^, n (%)	42 (35.0%)	–
BMI (kg/m^2^)	27.8 ± 2.4	< 26 kg/m^2^
Waist circumference (cm)	95.1 ± 6.9	< 100 cm for man < 90 cm for woman
Waist-to-hip ratio	0.92 ± 0.05	< 0.95 for man < 0.80 for woman
SBP (mmHg)	125.9 ± 13.1	< 130 mmHg
DBP (mmHg)	77.1 ± 9.7	< 90 mmHg
Triglyceride (mg/dl)	123.7 ± 70.5	< 150 mg/dl
LDL-C (mg/dl)	118.5 ± 27.6	< 100 mg/dl (optimal)
HDL-C (mg/dl)	55.7 ± 11.3	< 5 0 mg/dl for man < 40 mg/dl for woman
TG/HDL-C ratio	2.39 ± 1.61	< 2.0
AST (IU/l)	25.0 ± 9.6	< 40 IU/l
ALT (IU/l)	27.2 ± 18.3	< 40 IU/l
Apo A1^†^ (mg/dl)	142.5 (132–156.5)	75–160 mg/dl for man 80–175 mg/dl for woman
Apo B^†^ (mg/dl)	99.0 (85.5–112.0)	66–133 mg/dl for man 60–117 mg/dl
ApoB/A1 ratio^†^	0.70 (0.56–0.81)	< 0.77 for man < 0.63 for woman
Adiponectin^†^ (μg/dl)	6.6 (4.0–8.2)	5-37 μg/ml
Resistin^†^ (ng/dl)	5.3 (3.9–7.5)	–
Leptin^†^ (ng/dl)	28.94 (22.52–40.16)	< 12.5 ng/ml for man < 15.2 ng/ml for woman
Serotonin^†^ (ng/dl)	124.95 (93.65–172.7)	50–200 ng/ml
Fasting blood sugar^†^ (mg/dl)	98.5 (91–105)	70–100 mg/dl
Fasting insulin^†^ (μU/ml)	6.0 (4.2–9.6)	< 25 μU/ml
HbA1c^†^ (%)	5.5 (5.3–5.8)	< 5.7%
HOMA-IR^†^	1.48 (0.93–2.40)	0–2
HOMA-β^†^	62.1 (41.1–102.9)	> 107
IL-6^†^ (pg/dl)	1.6 (1.05–2.70)	< 43.5 pg/ml
IL-1β^†^ (pg/dl)	1.8 (1.1–5.5)	0.5–12 pg/ml
Visceral fat area (cm^2^)	117.0 ± 39.8	–
Subcutaneous fat area (cm^2^)	275.9 ± 73.3	–
Visceral fat/total body fat (%)	30.15 ± 8.60	–
Body fat mass (kg)	28.7 ± 4.6	–
Body fat percentage (%)	40.6 ± 4.5	–
Lean body mass (kg)	40.3 ± 8.8	–
Energy intake (kcal/day)	1474.3 ± 359.1	–
Carbohydrate intake (g/day)	207.4 ± 53.6	–
Lipid intake (g/day)	43.9 ± 15.5	–
Protein intake (g/day)	54.3 ± 16.3	–
Fiber intake (g/day)	15.8 ± 5.4	–
Folate intake (μg/day)	108.5 ± 67.8	–
Calcium intake (mg/day)	378.1 ± 133.8	–
Total cholesterol intake (mg/day)	207.6 ± 107.6	–
Saturated fatty acid intake (g/day)	8.59 ± 5.80	–
Polyunsaturated fatty acid intake (g/day)	10.1 ± 5.2	–

*BMI* body mass index, *SBP* systolic blood pressure, *DBP* diastolic blood pressure, *LDL-C* low-density lipoprotein cholesterol, *HDL-C* high-density lipoprotein cholesterol, *TG* triglyceride, *AST* aspartate aminotransferase, *ALT* alanine aminotransferase, *ApoA1* apolipoprotein A1, *ApoB* apolipoprotein B, *HOMA-IR* Homeostatic Model Assessment for Insulin Resistance, *HOMA-β* Homeostasis model assessment of β-cell function, *IL* interleukin.

^†^Phenotypes not following normal distribution are expressed as median (IQR).

^††^Alcohol consumption includes both current and past alcohol consumption.

The mean serum triglyceride (TG), low-density lipoprotein cholesterol (LDL-C), and high-density lipoprotein cholesterol (HDL-C) levels were 123.7 mg/dl, 118.5 mg/dl, and 55.7 mg/dl, respectively. The median apolipoprotein-A1, and apolipoprotein-B levels were measured to be 142.5 mg/dl and 99 mg/dl, respectively. Body fat composition was measured and calculated using CT and dual energy X-ray absorptiometry (DEXA). The visceral fat area, subcutaneous fat area, and the visceral to total body fat percentage were calculated to be 117.0 cm^2^, 275.9 cm^2^, and 30.15% on the median, respectively. Mean body fat mass measured by DEXA was 28.7 kg and mean body fat percentage was 40.6%.

We analysed the correlation between different phenotypic, inflammatory parameters (Supp. Figure 1). The BMI level shows a positive correlation with visceral fat area, subcutaneous fat area, waist circumference, and waist-to-hip ratio. The BMI also positively correlates with levels of IL-1β, IL-6, TG, and TG/HDL-C ratio. In contrast, HDL-C, and Apoprotein-A1 levels inversely correlates with apolipoprotein-B/apolipoprotein-A1 ratio, TG/HDL-C ratio, and BMI level (all *p* < 0.05 by Spearman’s rank correlation test).

### EV-derived microbiota composition

**Figure 1:**
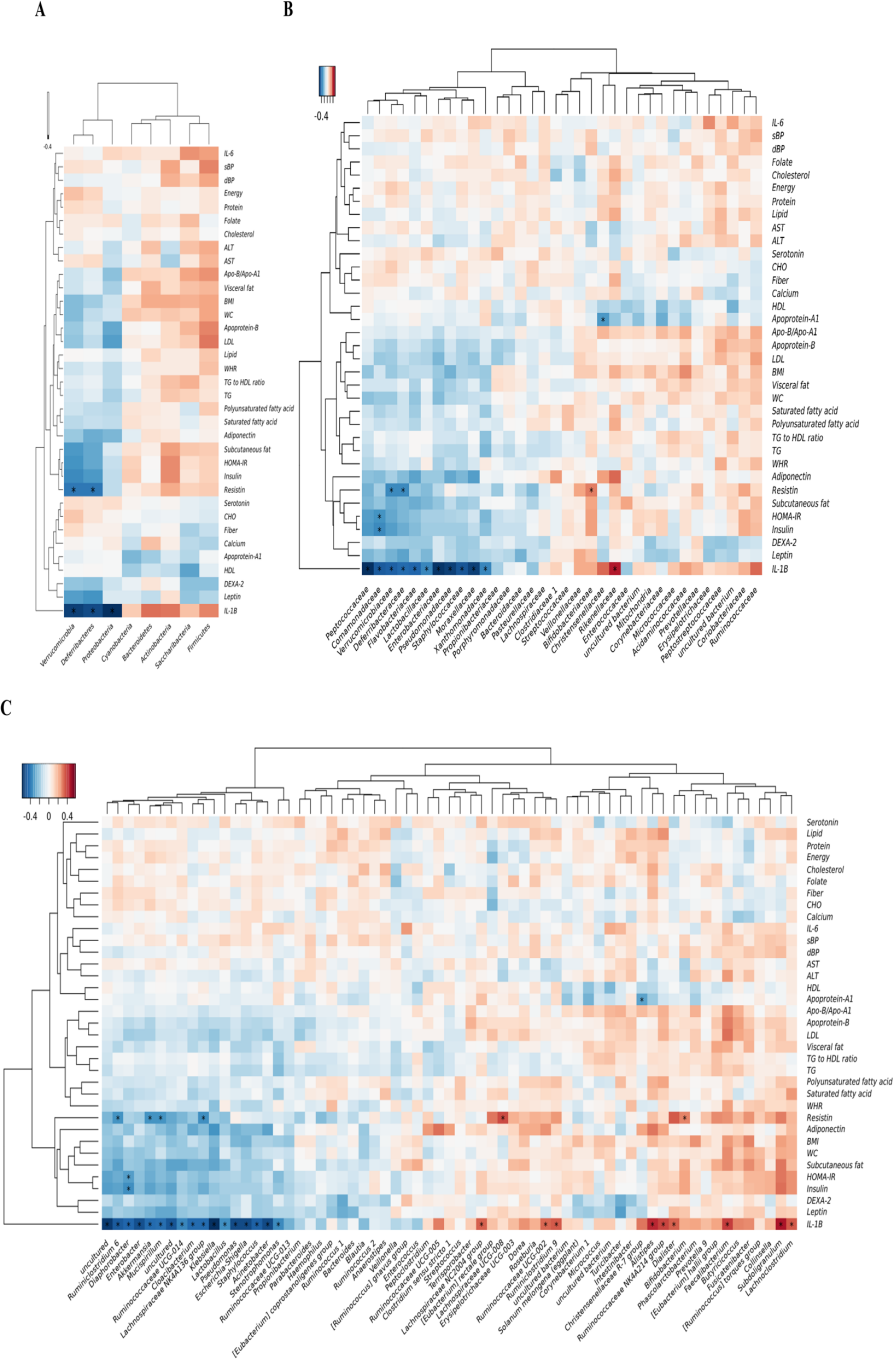
Correlation between clinical/laboratory parameters and microbial abundances. Correlation between different clinical parameters were analyzed by Spearman’s rank correlation analysis. Statistically significant correlation with FDR q value < 0.05 are indicated by asterisks. (**A**) Correlation in phylum level, (**B**) Correlation in family level. (**C**) Correlation in genus level. Dendrograms on X, Y axis were generated using complete-linkage hierarchical clustering. *WC* waist circumference, *BMI* body mass index, *IL-1B* interleukin-1β, *WHR* waist-to-hip ratio, *dBP* diastolic blood pressure, *sBP* systolic blood pressure, *ALT* alanine aminotransferase, *AST* aspartate aminotransferase, *TG* triglyceride, *HDL-C* high-density lipoprotein, *LDL-C* low-density lipoprotein, *IL-6* interleukin-6.

The faecal microbiome originating from bacterial cells and extracellular vesicles (EV) was profiled using 16S rDNA sequencing. There were no significant findings when analysing the microbiome originating from bacterial cells (data not shown). In contrast, when the correlation between faecal bacterial EV-derived microbiome composition and host phenotype was analysed, there were some significant correlations as the following (Fig. 1A–C). Clinical phenotypes, including serum level of IL-1β and resistin showed, significant correlation with the abundance of different faecal EV-derived microbiota of different species. A list of significant correlations between taxa and clinical variables with *p* < 0.05 is provided in Supplementary Table 1. Some of them are as follows: on the phylum level, the abundance of Firmicutes-derived EVs showed positive correlations with visceral fat area, serum apolipoprotein-B/apolipoprotein-A1 ratio, apolipoprotein-B, serum LDL-C level and serum IL-1β level (Spearman’s rank correlation coefficient ρ = 0.18, 0.19, 0.22, 0.24 and 0.24 respectively, all *p* < 0.05). On the genus level, the abundance of *Akkermansia*-derived EVs showed negative correlations with BMI and subcutaneous fat area (ρ = − 0.19, − 0.23 respectively, all *p* < 0.05). The abundance of *Akkermansia*-derived EVs also showed a negative correlation with serum IL-1β, leptin, fasting insulin, HOMA-IR, and resistin level (ρ = − 0.44, − 0.21, − 0.23, − 0.24 and − 0.32 respectively, all *p* < 0.05). The abundance of *Bacteroides*-derived EVs also showed weak positive correlations with serum leptin level (ρ = − 0.23, *p* = 0.01). The abundance of *Prevotella*-derived EVs also showed weak positive correlations with serum IL-1β level (ρ = 0.19, *p* = 0.04). Among them, however, only negative correlations between the abundance of EV-derived *Akkermansia,* and serum resistin and IL-1β levels remained significant after FDR adjustment (FDR q < 0.01).

### Gut microbe-derived extracellular vesicles in different enterotypes

**Figure 2:**
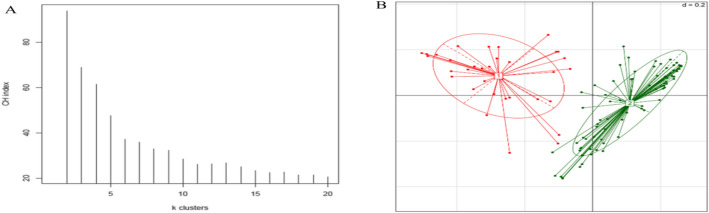
Enterotyping of the study subjects by extracellular vesicle-derived microbial compositions. (**A**) Calinski-Harabasz (CH) index. (**B**) Principal coordination analysis (PCoA) plot showing enterotype distribution of the obese population by Jensen–Shannon Divergence distance. The distance of one grid corresponds to 0.2 in Jensen–Shannon Divergence distance (d = 0.2).

The overall study population could be classified into two distinct enterotypes based on their stool EV-derived microbiome profile (enterotype I: Prevotellaceae-predominant, enterotype II: *Akkermansia/Bacteroides-*predominant, Fig. 2). The Calinski-Harabasz (CH) index was used to calculate the optimal number of clusters (Fig. 2A). Out of a total of 120 subjects, 34 were classified as enterotype I, and 86 were classified as enterotype II. In contrast, the bacterial cell-derived microbial compositions failed to separate the study population into distinct subgroups of patients (Supp. Figure 2A–C).

**Figure 3:**
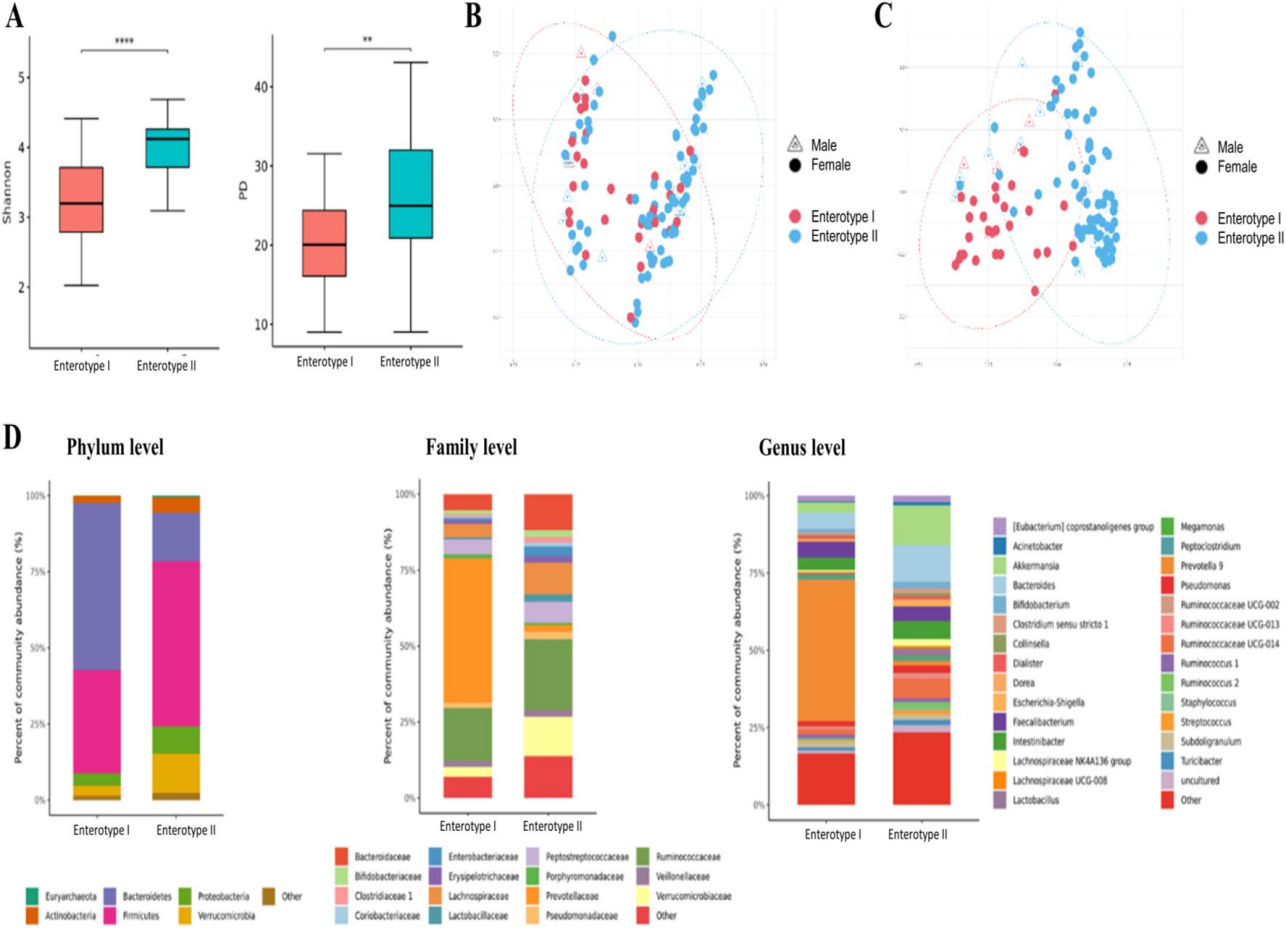
Extracellular vesicle-derived microbial diversity (**A**), β-diversity (principal coordinates analysis plots: **B**,**C**), and relative abundances (**D**) according to the enterotypes. (**A**) Shannon index and Faith’s phylogenetic diversity (Wilcoxon rank-sum test, *p* < 0.001, *p* = 0.003 for Shannon index and Faith’s phylogenetic diversity). Asterisks are added for *p* value < 0.05 (ns: *p* > 0.05, *p < 0.05, **p < 0.01, ***p < 0.001, ****p < 0.0001). (**B**) Principal coordinates analysis plots showing EV-derived bacterial distribution in two enterotypes by unweighted UniFrac distance. (PERMANOVA *p* = 0.463 by sex, *p* = 0.001 by enterotype) (**C**) Principal coordinates analysis plots showing EV-derived bacterial distribution in two enterotypes by weighted UniFrac distance. (PERMANOVA *p* = 0.022 by sex, *p* = 0.001 by enterotype) (**D**) Relative abundance of EV-derived microbiome (Phylum, Family and Genus levels).

We compared the species richness and evenness of the bacterial EV-derived microbiome between the two enterotype groups (Fig. 3A). The microbial diversity calculated by the Shannon index and Faith’s phylogenetic diversity were both significantly lower in enterotype I than in enterotype II (*p* < 0.001 and *p* = 0.003, respectively). The microbial composition, analysed by unweighted and weighted UniFrac distance, is depicted in Fig. 3B,C. Statistical analysis revealed a distinct distribution between the two enterotypes (PERMANOVA, all *p* = 0.001).

We analysed the relative abundance of gut microbe-derived EVs at the phylum, family, and genus levels (Fig. 3D, Supp. Table 2). Enterotype I subject showed significant enrichment of Bacteroidetes-derived EVs, and depletion of Actinobacteria, Firmicutes and Proteobacteria-derived EVs in phylum level (all FDR q < 0.05). Among the phylum Bacteroidetes, subjects in enterotype I showed a higher abundance of Prevotellaceae-derived EVs at the family level and *Prevotella*-derived EVs at the genus level (all FDR q < 0.05)*.* At the family level, subject in enterotype II had a higher abundance of Lachnospiraceae and Ruminococcaceae-derived EVs (all FDR q < 0.05). At the genus level, subjects in enterotype II a had significantly higher abundance of *Akkermansia-*derived EVs (FDR q < 0.05).

### Enterotype and host metabolic and inflammatory markers

The metabolic and inflammatory markers as well as the body fat compositions according to the enterotypes are summarised in Table 2. The enterotypes were independent of patient age and sex. Subjects in enterotype I tended to have higher levels of BMI, which did not reach statistical significance (*p* = 0.060).

**Table 2:** Characteristics of the subjects according to enterotypes.

Variables	Enterotype I (n = 34)	Enterotype II (n = 86)	*p* value	FDR q value
Sex, n (%)				
Male	5 (14.7)	16 (18.6)	0.810	0.966
Female	29 (85.3)	70 (81.4)		
Age (years)	45.6 ± 9.1	43.3 ± 8.8	0.211	0.844
Smoker, n (%)	3 (8.8)	4 (4.7)	0.655	0.966
Consumes alcohol^††^, n (%)	12 (35.3)	30 (34.9)	0.966	0.966
BMI (kg/m^2^)	28.4 ± 2.7	27.5 ± 2.2	0.060	0.300
Waist circumference (cm)	98.5 ± 8.1	94.6 ± 6.3	0.177	0.443
Waist-to-hip ratio	0.9 ± 0.1	0.9 ± 0.0	0.446	0.519
sBP (mmHg)	127.5 ± 12.8	125.9 ± 12.6	0.519	0.519
dBP (mmHg)	78.8 ± 10.0	77.2 ± 11.1	0.453	0.519
Triglyceride (mg/dl)	133.4 ± 69.1	119.8 ± 71.1	0.345	0.505
LDL-C (mg/dl)	117.9 ± 28.6	118.7 ± 27.3	0.876	0.876
HDL-C (mg/dl)	54.2 ± 13.5	56.3 ± 10.4	0.379	0.505
TG/HDL-C	2.8 ± 2.1	2.2 ± 1.4	0.130	0.505
AST (IU/ml)	26.3 ± 11.4	24.5 ± 8.8	0.362	0.362
ALT (IU/ml)	31.3 ± 20.1	25.5 ± 17.5	0.122	0.244
Apo A1^†^ (mg/dl)	139.5 (128.0–154.0)	143.0 (133.0–158.0)	0.344	0.833
Apo B^†^ (mg/dl)	97.0 (91.0–112.0)	99.0 (85.0–111.0)	0.818	0.956
ApoB/A1 ratio^†^	0.7 (0.6–0.9)	0.7 (0.6–0.8)	0.452	0.833
Adiponectin^†^ (μg/dl)	6.7 (4.0–8.3)	6.5 (4.0–8.2)	0.928	0.956
Resistin^†^ (ng/dl)	6.2 (4.3–9.3)	5.2 (3.8–7.1)	0.096	0.576
Leptin^†^ (ng/dl)	31.8 (22.7–40.2)	28.6 (22.5–40.1)	0.486	0.833
Serotonin^†^ (ng/dl)	123.2 (96.9–176.1)	125.5 (92.4–172.6)	0.956	0.956
Fasting blood sugar^†^ (mg/dl)	100 (90.5–105)	98 (92–106)	0.845	0.956
Fasting insulin^†^ (μU/ml)	7.4 (4.0–10.4)	6.0 (4.3–9.3)	0.623	0.935
HbA1c^†^ (%)	5.6 (5.4–6.0)	5.5 (5.3–5.7)	0.066	0.576
HOMA-IR^†^	1.6 (1.0–2.5)	1.4 (0.9–2.3)	0.458	0.833
HOMA-β^†^	71.0 (44.2–111.6)	60.1 (36–97.1)	0.331	0.833
IL-6^†^ (pg/dl)	1.6 (1.0–2.7)	1.6 (1.1–2.8)	0.622	0.622
IL-1β^†^ (pg/dl)	3.0 (1.3–5.7)	1.7 (1.1–5.3)	**0.025**	**0.050**
Visceral fat area (cm^2^)	124.0 ± 45.7	114.2 ± 37.0	0.227	0.341
Subcutaneous fat area (cm^2^)	292.7 ± 80.8	269.2 ± 69.4	0.116	0.232
Visceral fat/total body fat (%)	29.72 ± 8.29	30.32 ± 8.71	0.735	0.882
Total fat mass (kg)	30.12 ± 5.98	28.13 ± 5.05	0.068	0.232
Body fat percentage (%)	41.6 ± 3.7	40.1 ± 4.8	0.106	0.232
Lean body mass (kg)	40.36 ± 8.69	40.22 ± 8.90	0.939	0.939
Dietary intake				
Energy intake (kcal/day)	1409.9 ± 287.6	1499.4 ± 381.8	0.226	0.863
Carbohydrate intake (g/day)	198.7 ± 41.2	210.7 ± 57.6	0.207	0.863
Lipid intake (g/day)	42.8 ± 15.0	44.3 ± 15.8	0.645	0.880
Protein intake (g/day)	52.0 ± 11.8	55.2 ± 17.7	0.259	0.886
Fiber intake (g/day)	15.5 ± 5.1	15.9 ± 5.6	0.758	0.880
Folate intake (μg/day)	108.8 ± 68.0	108.4 ± 68.1	0.980	0.980
Calcium intake (mg/day)	393.9 ± 138.5	371.9 ± 132.2	0.424	0.880
Total cholesterol intake (mg/day)	202.9 ± 94.5	209.4 ± 112.8	0.771	0.880
Saturated fatty acid intake (g/day)	9.0 ± 6.1	8.4 ± 5.7	0.667	0.880
Polyunsaturated fatty acid intake (g/day)	10.3 ± 4.3	10.0 ± 5.5	0.792	0.880

Categorical variables were expressed as number of subjects (n), (%), and compared using χ^2^-test. Continuous variables are expressed as mean ± standard deviation, and compared using Student’s t-test, unless otherwise noted.

The FDR is calculated by adjusting raw *p* values with Benjamini–Hochberg method.

*FDR* false discovery rate, *BMI* body mass index, *sBP* systolic blood pressure, *dBP* diastolic blood pressure, *LDL-C* low-density lipoprotein cholesterol, *HDL-C* high-density lipoprotein cholesterol, *TG* triglyceride, *AST* aspartate aminotransferase, *ALT* alanine aminotransferase, *ApoA1* apolipoprotein A1, *ApoB* apolipoprotein B, *HOMA-IR* Homeostatic Model Assessment for Insulin Resistance, *HOMA-β* Homeostasis model assessment of β-cell function, *IL-6* interleukin-6, *IL-1β* interleukin-Iβ, *IQR* interquartile range.

Bold style indicates statistical significance.

^†^Phenotypes not following normal distribution were compared using non-parametric analysis: Wilcoxon rank-sum test. These variables are expressed as median (IQR).

^††^Alcohol consumption includes both current and past alcohol consumption.

Although no significant difference was seen, serum resistin tended to be higher in subjects in enterotype I compared to subjects in enterotype II (nominal *p* = 0.096). Serum IL-1β levels were higher in subjects in enterotype I than in those in enterotype II (nominal *p* = 0.025 and FDR q = 0.050). In contrast, there was no significant difference in serum IL-6 levels between the two enterotype groups (*p* = 0.622).

The total body fat mass also tended to be higher in the enterotype I group than in enterotype II group (nominal *p* = 0.068). Both visceral fat area and subcutaneous fat area did not differ between the two enterotypes. There was no difference in the dietary intake of total calories, carbohydrates, lipids, proteins, fibers, or total cholesterols.

Further analysis on the clinical variables were performed according to sex (Supp. Table 3). The distribution of overall anthropometric measurements, metabolic parameters, and inflammatory parameters by enterotype showed generally similar trends in both men and women. In men, however, BMI and waist circumference were significantly lower in enterotype II (nominal *p* = 0.002 and 0.028, respectively, Supp. Table 3), and HDL-C level were significantly higher in enterotype II (nominal *p* = 0.024, Supp. Table 3). In women, BMI and waist circumference failed to show statistically significant difference (*p* > 0.05, Supp. Table 3).

**Figure 4:**
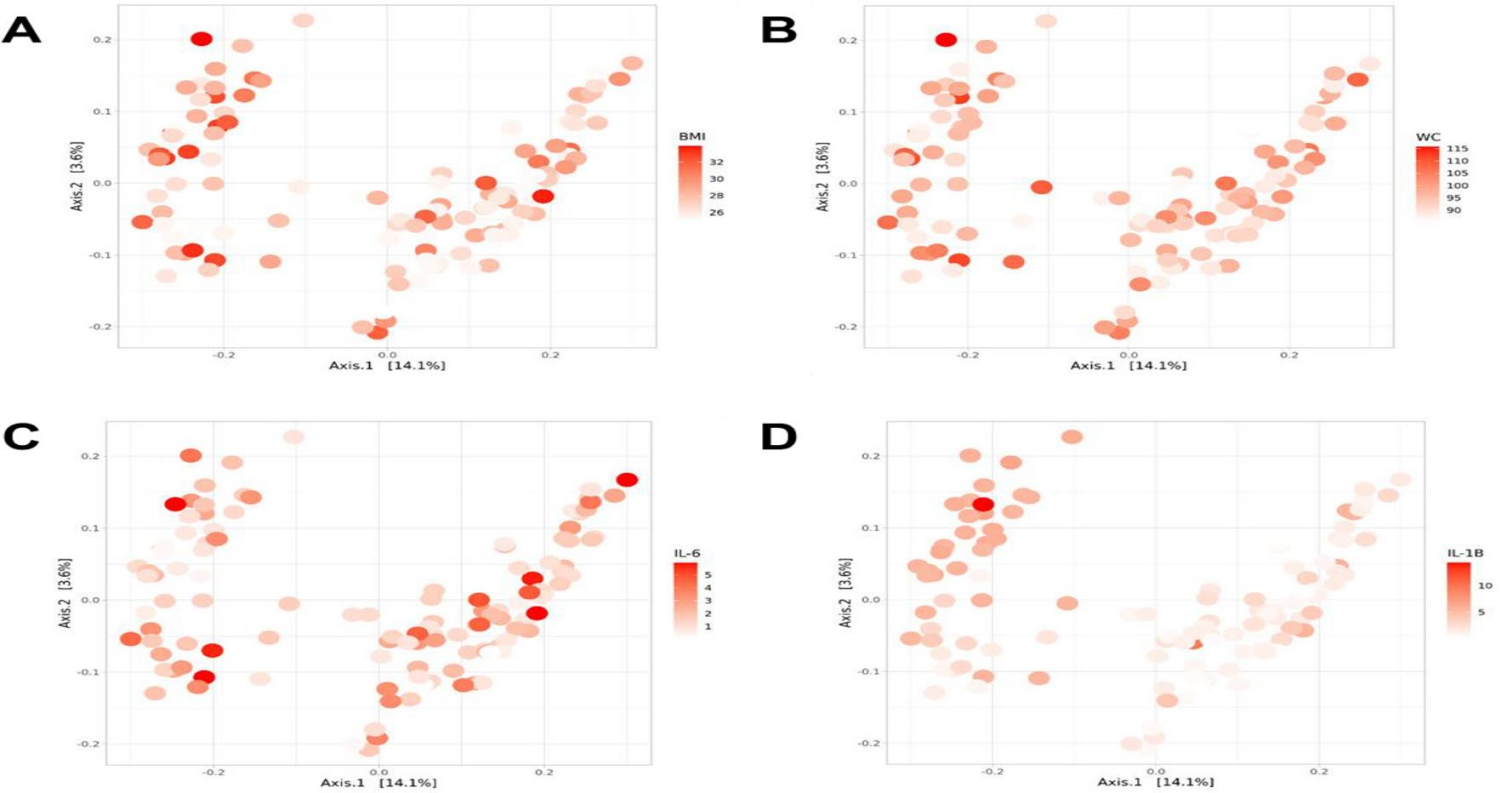
EV-derived microbiome profile and distribution of clinical parameters and inflammatory markers in MHO subjects. (**A**) Weighted UniFrac distance matrix showing distribution of body mass index. (**B**) Weighted UniFrac distance matrix showing distribution of waist circumference. (**C**) Weighted UniFrac distance matrix showing distribution of interleukin-6. (**D**) Weighted UniFrac distance matrix showing distribution of interleukin-1β.

The distribution of some phenotypic and inflammatory markers in faecal EV-derived microbiome is visually depicted in Fig. 4. The microbiome profile of the study participants appears to be largely divided into two clusters. Interestingly, the distribution of faecal EV-derived microbiome profiles according to serum IL-1β levels appeared to be markedly different in the two clusters (Fig. 4D). The distribution of faecal EV-derived microbiome profiles did not differ according to the BMI (Fig. 4A), waist circumference (Fig. 4B) or serum IL-6 levels in the two enterotypes (Fig. 4C).

In the case of the bacterial cell-derived microbiome, there were no significant findings based on anthropometric and inflammatory biomarkers of obesity (Supp. Figure 3A–D).

## Discussion

In this cross-sectional study, we have shown the characteristics of faecal EV-derived microbial composition in metabolically healthy obese individuals. For example, the abundance of *Akkermansia*-derived EVs negatively correlated with subcutaneous fat area as well as total BMI; they also negatively correlated with serum IL-1β, leptin, fasting insulin, HOMA-IR and resistin levels (Fig. 1, Supplementary Table 1). On the other hand, the abundance of *Prevotella*-derived EVs positively correlated with serum IL-1β levels.

The gut *Akkermansia* is reported to be associated with a healthier clinical profile, and its abundance is decreased in obese patients^27–29^. The anti-inflammatory effects of *Akkermansia*-derived EVs have been previously reported in various studies^22–24^. *A. muciniphila* is known to enhance gut epithelial barrier function and shows anti-inflammatory effects^30–32^. Our findings are consistent with these previous studies in that the abundance of *Akkermansia*-derived EVs negatively correlates with BMI and serum levels of inflammatory cytokines.

On the contrary, the relative abundance of *Prevotella* has been reported to be associated with increasing BMI in obese patients^16,28,33^. Conying Chen et al. have reported an increased relative abundance of *P. copri* in obese pig models, compared to that of non-obese pigs, and the abundance of *P. copri* was associated with the serum metabolite levels associated with obesity^34^. Similary, De Vadder et al. have used germ-free mice and reported that the host chronic inflammatory response deteriorated in *P. copri*-gavaged mice. The study also revealed that succinate produced by *P. copri* improves glucose metabolism and insulin sensitivity in obese mice^35^.

In this study, the study participants were a relatively homogenous group of obese individuals with no metabolic or cardiovascular comorbidities. The faecal bacterial cell-derived microbial composition did not show any distinction in the overall study population. However, the study subjects could be grouped into two distinct groups, called enterotypes, based on their faecal bacterial EV-derived microbiome composition. The faecal EV-derived microbial composition of the two enterotypes showed significant differences in the relative abundance of *Prevotella* and *Akkermansia*-derived EVs.

This result is consistent with previous studies on the gut microbiome of obese patients. Lars Christensen et al*.* reported that obese people can be categorised into two groups by the gut microbiome^36^. Manimozhiyan Arumugam et al*.* also reported distinct enterotype groups in obese subjects, and *Bacteroides*, *Prevotella*, and *Ruminococcus* were the main contributors to the differentiation between the two enterotypes^37^. Lars Christensen et al. has reported that *Prevotella-*rich group is associated with high dietary carbohydrate, resistant starch, and fiber. *Bacteroides*-rich enterotype was associated with high dietary fat and low dietary fiber. In this study, patient reported dietary intake of carbohydrate, fiber and fat content failed to show significant difference between the two enterotypes. The negative results of our study might be attributed to the relatively small sample size of the study. Another possible reason could be a recall bias as we investigated dietary intake using self-reported survey results.

Previous studies have demonstrated that the gut microbiome may influence the metabolic health of the host through various interactions. Bacterial cell-derived microbiome includes DNAs from dead or inactive bacteria as well as living bacteria. In contrast, analysis of bacterial DNA in the EVs are believed to reflect the actual activity of bacteria in the faecal material^38,39^. Recent studies have provided evidence that the EVs from the gut microbiome play an important role in the interaction between the gut microbiome and host metabolism^20,22^. It has been reported that EV-derived microbial composition, rather than faecal bacterial DNA, is more representative of actual bacterial activity and its impact on host health conditions^40–42^. Nevertheless, there are not enough studies on how EV-derived 16S ribosomal DNA profiles are associated with obesity or related metabolic complications. In this study, the microbial composition analysis of the bacterial cell-derived microbiome did not show definite clustering (Supp. Fig S2), whereas EV-derived microbial composition can be used to discriminate the obese population into two groups.

The differences in clinical parameters, including inflammatory cytokines and body fat composition, between these two enterotypes were also analysed in this study. Interestingly, the two enterotypes showed significant differences in serum IL-Iβ levels. IL-Iβ is an inflammatory cytokine that is activated by inflammasomes. The activation of IL-Iβ is known to be a key process that contribute to the initiation of insulin resistance and type 2 diabetes^43^. Moreover, evidence suggests that the inflammasome and IL-Iβ are linked to obesity-related diseases^44,45^. While no other clinical parameters showed significant difference between the two enterotypes, this preliminary results, difference in IL-1β might imply possibility that the two enterotypes might have different systemic inflammatory, metabolic state, and thereby resulting in difference in long-term clinical outcomes.

Interestingly, when analysing metabolic and inflammatory parameters according to the enterotype, some differences were observed by sex. Although trends in metabolic and inflammatory parameters were similar in both sex groups, BMI, waist circumference, and HDL-C levels showed statistical differences only in men. Additionally, a few clinical parameters, apolipoprotein-B, HOMA-IR, and visceral/total body fat ratio might have different distribution by sex, but all of them failed to show statistical significance to draw any conclusion. The physiologic difference in the sex hormone levels and body fat composition between men and women may contribute to the differences in clinical parameters and EV-derived microbiome by sex. However, our study lacked in the number of subjects to perform any further subgroup analyses, thus further studies with larger number of subjects may be able to elucidate the difference in men and pre/post-menopausal women.

The limitations of this study must be acknowledged. First, multiple testing on serum IL-1β levels between the two enterotypes revealed an FDR q-value of 0.050. This lack of statistically significant results might be attributed to the limited number of study subjects enrolled in the study, which limits generalisation of the result or further subgroup analysis. Second, our findings suggest that EV-derived microbial composition and metabolic parameters might have different clinical implications in male and female groups. However, our study population had sex discrepancies and there were not enough male subjects to perform additional subgroup analysis by sex. Furthermore, the cross-sectional study design could not show the impact of different EV-derived microbial compositions on short-term, or long-term prognosis. Further studies with larger number of obese patients and long-term follow-up period would further clarify the correlation between different metabolic conditions and EV-derived microbial composition.

In conclusion, the EV-derived microbial abundance shows association with different anthropometric, metabolic, and inflammatory parameters in metabolically healthy obese subjects. Our findings show that MHO individuals can be categorised into two discrete groups based on their faecal bacterial EV-derived microbial composition. The two enterotypes may have difference in their IL-1β levels, but did not show statistical differences in other anthropometric, metabolic, or inflammatory parameters. Although this study results remain inconclusive, it suggests the possibility to uncover relationships between EV-derived microbiomes and metabolic parameters or long-term outcomes in MHO subjects. Further studies with larger number of subjects and analysis might elucidate the impact of EV-derived microbial composition on metabolic parameters or long-term prognosis in MHO subjects.

## Method

### Patients and sample collection

This study was conducted using MHO subjects enrolled in a randomised, double-blind, placebo-controlled clinical trial to evaluate the efficacy of green tea and fermented green tea extract (Clinical Trial No: NCT03537625). In this study, we have defined MHO by the following criteria: (1) systolic blood pressure less than 130 mm Hg and no use of blood pressure–lowering medication, (2) waist-to-hip ratio (WHR) less than 0.95 (women) and less than 1.03 (men), and (3) no prevalent type 2 diabetes^46^. Baseline demographic data, past medical history, smoking habits, drinking habits and dietary/exercise habits of the study subjects were collected using patient-reported surveys and questionnaires, and body composition measurements were performed using DEXA scans. Faecal and blood samples were collected on the second visit, following the screening period. This study was approved by the Institutional Review Board of Seoul National University Bundang Hospital (IRB No: B-1604/342-005) Written informed consent was obtained from all study participants.

### Extracellular vesicles (EV) isolation and DNA extraction

Human stool samples were diluted in 10 mL of PBS and filtered through a cell strainer. EVs were separated from the filtered samples by centrifuging the solution at 10,000×*g* for 10 min at 4 °C. The EVs were suspended in the supernatant of the samples, and the bacterial cells were concentrated in the pellets. The supernatants were then filtered through a 0.22-μm filter to eliminate bacterial and foreign particles from the stool samples. EVs were heated at 100 °C for 40 min to extract genomic material from the bacterial cells and EVs, and centrifuged for 30 min at 13,000 rpm at 4 °C to remove the remaining foreign particles and wastes. The DNeasy PowerSoil Kit (QIAGEN, Germany) was used to extract DNA from the samples, which was quantified using a QIAxpert system (QIAGEN, Germany).

### Bacterial metagenomic analysis using extracellular vesicles (EV) DNA

Bacterial genomic DNA was amplified with primers specific for the V3–V4 regions of the 16S rRNA gene, 16S_V3_F (5′-TCGTCGGCAGCGTCAGATGTGTATAAGAGACAGCCTACGGGNGGCWGCAG-3′) and 16S_V4_R (5′-TCTCGTGGGCTCGGAGATGTGTATAAGAGACAGGACTACHVGGGTATCTAATCC-3′). Libraries were prepared using PCR products according to the MiSeq System guide (Illumina, USA) and quantified using QIAxpert (QIAGEN, Germany). Each amplicon was quantified, set to an equimolar ratio, pooled, and sequenced on a MiSeq platform (Illumina, USA) according to the recommendations of the manufacturer.

### Analysis of bacterial composition

Paired-end reads that matched the adapter sequences were trimmed using Cutadapt version 1.1.6, with a minimum overlap, a maximum error rate, and a minimum length of 11, 15% and 10, respectively. The resulting FASTQ files containing paired-end reads were merged with CASPER version 0.8.2, with a mismatch ratio of 0.27, and then quality-filtered using the Phred (Q) score-based criteria. Any reads shorter than 350 bp or longer than 550 bp after merging were discarded. A reference-based chimera detection step was performed using VSEARCH version 2.3.0 against the SILVA gold database to identify the chimeric sequences. Sequence reads were clustered into operational taxonomic units (OTUs) using VSEARCH with an open clustering algorithm at a 97% sequence similarity threshold. The representative sequences of the OTUs were classified using the SILVA 132 database with UCLUST (parallel_assign_taxonomy_uclust.py script in QIIME version 1.9.1) under default parameters. The original contributions presented in this study are publicly available in NCBI database. Raw reads of the faecal microbiota and microbe-derived EVs for obese subjects were deposited into the NCBI SRA database (Accession Numbers: SRR15182562–SRR15182631; SRR15182632–SRR15182701).

### Enterotyping

In both study sets, enterotyping was performed using R (EMBL3). In brief, the Jensen–Shannon divergence of the samples was calculated based on the family-level bacterial compositions of the subjects. Partitioning around medoids clustering was performed based on the distance matrix. The optimal number of clusters was chosen by maximising the Calinski–Harabasz index (‘index.G1’ function in the R library ‘clusterSim’). The result of clustering was visualised with a PCA plot using the ‘s.class’ function in the R ade4 package.

### Statistical analysis

Bacterial composition and diversity was analysed and graphed using Qiime2 (2021-11) and R (version 3.6.3). Microbes meeting two filtering criteria for inclusion in the analysis were selected: (1) a minimum abundance of 0.05% across the entire dataset and (2) presence in more than 50% of the individuals. α-Diversity (observed OTUs, Shannon index, and phylogenetic diversity) was compared by the decimal log-transformed relative abundance of faecal microbiota between groups using the Wilcoxon rank-sum test (R package ‘microbiome version 1.9.19’). The FDR was calculated by adjusting raw *p* values with Benjamini–Hochberg method.

Group distances for β-diversity (weighted UniFrac metric and unweighted UniFrac metric) were generated with permutational analysis of variance (PERMANOVA) using 1000 Monte Carlo permutations (R package ‘phyloseq version 1.30.0’ and ‘vegan version 2.5.6’) and visualised using principal coordinate analysis (PCoA) plots.

For the clinical phenotypes whose distribution followed normal distribution, the differences in the two enterotypes were analysed using Student’s t-test. For the phenotypes that did not follow normal distribution, the differences in the two enterotypes were analysed by Wilcoxon rank-sum test. The correlation of clinical phenotypes and relative abundance of microbiota were analysed by Spearman’s rank correlation test. *p* values under 0.05 was considered statistically significant difference.

### Ethics approval and consent to participate

This study was performed in accordance with the Declaration of Helsinki. This study was approved by the Institutional Review Board of Seoul National University Bundang Hospital (IRB No: B-1604/342-005). All methods were carried out in accordance with the guidelines and regulations of ethics committee of Seoul National University Bundang Hospital. Written informed consent were obtained from all the study participants.

### Supplementary Information

Supplementary Tables.

Supplementary Figures.

## Data Availability

The dataset analysed during the current study are available in the BioProject repository, ID: PRJNA881023. Accession Numbers: SRR15182562–SRR15182631; SRR15182632–SRR15182701(https://www.ncbi.nlm.nih.gov/sra?LinkName=bioproject_sra_all&from_uid=881023).

## Abbreviations

ApoA1: Apolipoprotein A1
ApoB: Apolipoprotein B
ALT: Alanine aminotransferase
AST: Aspartate aminotransferase
BMI: Body mass index
CH index: Calinski–Harabasz index
dBP: Diastolic blood pressure
DEXA: Dual energy X-ray absorptiometry
EV: Extracellular vesicle
Faith’s PD: Faith’s phylogenetic diversity
HDL-C: High-density lipoprotein cholesterol
HOMA-IR: Homeostatic model assessment for insulin resistance
HOMA-β: Homeostasis model assessment of β-cell function
IL-1 β: Interleukin-1β
IL-6: Interleukin-6
LDL-C: Low-density lipoprotein cholesterol
MHO: Metabolically healthy obese
OTU: Operational taxonomic unit
PCoA: Principal coordinate analysis
sBP: Systolic blood pressure
TG: Triglyceride
WC: Waist circumference
WHR: Waist-to-hip ratio

## References

[1] Elías-López D (2021). Natural course of metabolically healthy phenotype and risk of developing Cardiometabolic diseases: A three years follow-up study. BMC Endocr. Disord..

[2] Apovian CM (2016). Obesity: Definition, comorbidities, causes, and burden. Am. J. Manage. Care.

[3] Federation, T. W. O. World_Obesity_Atlas_2022_WEB.pdf. (2022).

[4] Phillips CM (2013). Metabolically healthy obesity: Definitions, determinants and clinical implications. Rev. Endocr. Metab. Disord..

[5] Bell JA, Kivimaki M, Hamer M (2014). Metabolically healthy obesity and risk of incident type 2 diabetes: A meta-analysis of prospective cohort studies. Obes. Rev..

[6] Aung K, Lorenzo C, Hinojosa MA, Haffner SM (2014). Risk of developing diabetes and cardiovascular disease in metabolically unhealthy normal-weight and metabolically healthy obese individuals. J. Clin. Endocrinol. Metab..

[7] Eshtiaghi R, Keihani S, Hosseinpanah F, Barzin M, Azizi F (2015). Natural course of metabolically healthy abdominal obese adults after 10 years of follow-up: The Tehran Lipid and Glucose Study. Int. J. Obes. (Lond.).

[8] Sommer F, Bäckhed F (2013). The gut microbiota–masters of host development and physiology. Nat. Rev. Microbiol..

[9] Cuevas-Sierra A, Ramos-Lopez O, Riezu-Boj JI, Milagro FI, Martinez JA (2019). Diet, gut microbiota, and obesity: Links with host genetics and epigenetics and potential applications. Adv. Nutr..

[10] Alili R (2021). Characterization of the gut microbiota in individuals with overweight or obesity during a real-world weight loss dietary program: A focus on the bacteroides 2 enterotype. Biomedicines.

[11] Aron-Wisnewsky J (2019). Major microbiota dysbiosis in severe obesity: Fate after bariatric surgery. Gut.

[12] Cotillard A (2013). Dietary intervention impact on gut microbial gene richness. Nature.

[13] Vandeputte D (2017). Quantitative microbiome profiling links gut community variation to microbial load. Nature.

[14] Hjorth MF (2019). Prevotella-to-Bacteroides ratio predicts body weight and fat loss success on 24-week diets varying in macronutrient composition and dietary fiber: Results from a post-hoc analysis. Int. J. Obes. (Lond.).

[15] Magne F (2020). The firmicutes/bacteroidetes ratio: A relevant marker of gut dysbiosis in obese patients?. Nutrients.

[16] Duan M (2021). Characteristics of gut microbiota in people with obesity. PLoS One.

[17] Zhong X (2020). Gut microbiota associations with metabolic health and obesity status in older adults. Nutrients.

[18] Yáñez-Mó M (2015). Biological properties of extracellular vesicles and their physiological functions. J. Extracell. Vesicles.

[19] Díez-Sainz E, Milagro FI, Riezu-Boj JI, Lorente-Cebrián S (2021). Effects of gut microbiota-derived extracellular vesicles on obesity and diabetes and their potential modulation through diet. J. Physiol. Biochem..

[20] Woith E, Fuhrmann G, Melzig MF (2019). Extracellular vesicles-connecting kingdoms. Int. J. Mol. Sci..

[21] Song EJ (2020). Effect of probiotics on obesity-related markers per enterotype: A double-blind, placebo-controlled, randomized clinical trial. Epma J..

[22] Kang CS (2013). Extracellular vesicles derived from gut microbiota, especially *Akkermansia muciniphila*, protect the progression of dextran sulfate sodium-induced colitis. PLoS One.

[23] Ashrafian F (2019). Comparative study of effect of *Akkermansia*
*muciniphila* and its extracellular vesicles on toll-like receptors and tight junction. Gastroenterol. Hepatol. Bed Bench.

[24] Chelakkot C (2018). *Akkermansia muciniphila*-derived extracellular vesicles influence gut permeability through the regulation of tight junctions. Exp. Mol. Med..

[25] Shen Y (2012). Outer membrane vesicles of a human commensal mediate immune regulation and disease protection. Cell Host Microbe.

[26] Choi Y (2015). Gut microbe-derived extracellular vesicles induce insulin resistance, thereby impairing glucose metabolism in skeletal muscle. Sci. Rep..

[27] Dao MC (2019). *Akkermansia muciniphila* abundance is lower in severe obesity, but its increased level after bariatric surgery is not associated with metabolic health improvement. Am. J. Physiol. Endocrinol. Metab..

[28] Cani PD (2018). Human gut microbiome: Hopes, threats and promises. Gut.

[29] Li J (2017). Gut microbiota dysbiosis contributes to the development of hypertension. Microbiome.

[30] Anhê FF (2015). A polyphenol-rich cranberry extract protects from diet-induced obesity, insulin resistance and intestinal inflammation in association with increased *Akkermansia* spp. population in the gut microbiota of mice. Gut.

[31] Zhang X (2013). Human gut microbiota changes reveal the progression of glucose intolerance. PLoS One.

[32] Greer RL (2016). *Akkermansia muciniphila* mediates negative effects of IFNγ on glucose metabolism. Nat. Commun..

[33] Stanislawski MA, Dabelea D, Lange LA, Wagner BD, Lozupone CA (2019). Gut microbiota phenotypes of obesity. NPJ Biofilms Microbiomes.

[34] Chen C (2021). *Prevotella*
*copri* increases fat accumulation in pigs fed with formula diets. Microbiome.

[35] De Vadder F (2016). Microbiota-produced succinate improves glucose homeostasis via intestinal gluconeogenesis. Cell Metab..

[36] Christensen L, Roager HM, Astrup A, Hjorth MF (2018). Microbial enterotypes in personalized nutrition and obesity management. Am. J. Clin. Nutr..

[37] Arumugam M (2011). Enterotypes of the human gut microbiome. Nature.

[38] Choi DS, Kim DK, Kim YK, Gho YS (2015). Proteomics of extracellular vesicles: Exosomes and ectosomes. Mass Spectrom. Rev..

[39] Shin CM (2021). Validity and safety of ID-JPL934 in lower gastrointestinal symptom improvement. Sci. Rep..

[40] Park J (2021). Fecal microbiota and gut microbe-derived extracellular vesicles in colorectal cancer. Front Oncol..

[41] Yoon H (2023). Analysis of the gut microbiome using extracellular vesicles in the urine of patients with colorectal cancer. Korean J. Intern. Med..

[42] Heo M (2023). Potential of gut microbe-derived extracellular vesicles to differentiate inflammatory bowel disease patients from healthy controls. Gut Liver.

[43] Stienstra R (2010). The inflammasome-mediated caspase-1 activation controls adipocyte differentiation and insulin sensitivity. Cell Metab..

[44] Maedler K (2002). Glucose-induced beta cell production of IL-1beta contributes to glucotoxicity in human pancreatic islets. J. Clin. Invest..

[45] Febbraio MA (2014). Role of interleukins in obesity: Implications for metabolic disease. Trends Endocrinol. Metab..

[46] April-Sanders AK, Rodriguez CJ (2021). Metabolically healthy obesity redefined. JAMA Netw. Open.

