# Supplementary material for: Gut microbiome signature of metabolically healthy obese individuals according to anthropometric, metabolic and inflammatory parameters
Source: Sci Rep. 2024 Feb 11;14:3449. doi: 10.1038/s41598-024-53837-z (PMC10859373; doi:10.1038/s41598-024-53837-z)
Supplement: Supplementary file 1 — Supplementary Tables. [file 41598_2024_53837_MOESM1_ESM.docx]

**Supplementary table 1.** Correlation with clinical parameters and faecal EV-derived microbial abundance

|  | **Clinical phenotypes** | **ρ** | ***p*-value** | **FDR-q** |
| --- | --- | --- | --- | --- |
| **Phylum level** |  |  |  |  |
| Bacteroidetes | IL-1β | 0.2592 | 0.0043 | 0.1490 |
| Verrucomicrobia | BMI | -0.1887 | 0.0390 | 0.1949 |
|  | **Resistin** | -0.3158 | 0.0004 | 0.0077 |
|  | Leptin | -0.2053 | 0.0245 | 0.1428 |
|  | Insulin | -0.2293 | 0.0118 | 0.0978 |
|  | HOMA-IR | -0.2407 | 0.0086 | 0.0978 |
|  | **IL-1β** | **-0.4353** | **<0.0001** | **<0.0001** |
|  | Subcutaneous fat | -0.2258 | 0.0140 | 0.0978 |
| Saccharibacteria | HDL-C | -0.2508 | 0.0057 | 0.2002 |
|  | Leptin | -0.2241 | 0.0139 | 0.2425 |
|  | IL-6 | 0.1913 | 0.0363 | 0.3176 |
|  | DEXA-2 | -0.1937 | 0.0348 | 0.3176 |
| Actinobacteria | BMI | 0.1843 | 0.0439 | 0.2570 |
|  | AST | -0.1812 | 0.0476 | 0.2570 |
|  | Resistin | 0.1932 | 0.0345 | 0.2570 |
|  | Insulin | 0.1933 | 0.0344 | 0.2570 |
|  | IL-1β | 0.2210 | 0.0153 | 0.2570 |
|  | Subcutaneous fat | 0.2213 | 0.0160 | 0.2570 |
| Proteobacteria | LDL-C | -0.2483 | 0.0062 | 0.1093 |
|  | ApoB | -0.2294 | 0.0117 | 0.1369 |
|  | ApoB/ApoA1 | -0.1898 | 0.0379 | 0.2653 |
|  | Adiponectin | -0.2038 | 0.0256 | 0.2238 |
|  | **IL-1β** | **-0.4797** | **<0.0001** | **<0.0001** |
| Cyanobacteria | ApoA1 | -0.2050 | 0.0247 | 0.8060 |
| Firmicutes | sBP | 0.2507 | 0.0057 | 0.1232 |
|  | dBP | 0.2233 | 0.0142 | 0.1232 |
|  | LDL-C | 0.2359 | 0.0095 | 0.1232 |
|  | ApoB | 0.2164 | 0.0176 | 0.1232 |
|  | ApoB/ApoA1 | 0.1875 | 0.0403 | 0.1761 |
|  | IL-1β | 0.2189 | 0.0163 | 0.1232 |
|  | Visceral fat | 0.1834 | 0.0468 | 0.1820 |
|  | DEXA-2 | -0.2089 | 0.0226 | 0.1318 |
|  | Calcium intake | -0.1930 | 0.0364 | 0.1761 |
| Deferribacteres | Adiponectin | -0.2079 | 0.0227 | 0.1189 |
|  | **Resistin** | **-0.3308** | **0.0002** | **0.0039** |
|  | Leptin | -0.2340 | 0.0101 | 0.1179 |
|  | Insulin | -0.2063 | 0.0238 | 0.1189 |
|  | HOMA-IR | -0.2173 | 0.0181 | 0.1189 |
|  | **IL-1β** | **-0.4185** | **<0.0001** | **0.0001** |
|  | Subcutaneous fat | -0.2123 | 0.0210 | 0.1189 |
| **Family level** |  |  |  |  |
| Prevotellaceae | IL-1β | 0.1870 | 0.0408 | 0.6967 |
| Pasteurellaceae | Resistin | -0.2116 | 0.0204 | 0.3672 |
|  | IL-1β | -0.2106 | 0.0210 | 0.3672 |
|  | Subcutaneous fat | -0.1849 | 0.0450 | 0.5253 |
| Peptococcaceae | BMI | -0.2077 | 0.0228 | 0.1140 |
|  | WC | -0.2215 | 0.0150 | 0.1053 |
|  | Adiponectin | -0.1845 | 0.0437 | 0.1700 |
|  | Resistin | -0.2435 | 0.0074 | 0.0644 |
|  | Leptin | -0.1859 | 0.0420 | 0.1700 |
|  | Insulin | -0.2512 | 0.0057 | 0.0644 |
|  | **HOMA-IR** | **-0.2727** | **0.0028** | **0.0491** |
|  | **IL-1β** | **-0.5332** | **<0.0001** | **<0.0001** |
|  | Subcutaneous fat | -0.2114 | 0.0216 | 0.1140 |
| Staphylococcaceae | Adiponectin | -0.2008 | 0.0279 | 0.4240 |
|  | **IL-1β** | **-0.4679** | **<0.0001** | **<0.0001** |
| Pseudomonadaceae | BMI | -0.2536 | 0.0052 | 0.0607 |
|  | Adiponectin | -0.2670 | 0.0032 | 0.0560 |
|  | **IL-1β** | **-0.5087** | **<0.0001** | **<0.0001** |
| Rikenellaceae | BMI | 0.1804 | 0.0486 | 0.2127 |
|  | AST | -0.1884 | 0.0393 | 0.2127 |
|  | ApoA1 | -0.2202 | 0.0156 | 0.1763 |
|  | Adiponectin | 0.2700 | 0.0029 | 0.0501 |
|  | **IL-1β** | **0.4179** | **<0.0001** | **0.0001** |
|  | Lipid intake | 0.1895 | 0.0398 | 0.2127 |
|  | Cholesterol intake | 0.2137 | 0.0202 | 0.1763 |
|  | Polyunsaturated fatty acid intake | 0.1824 | 0.0481 | 0.2127 |
| Veillonellaceae | Resistin | 0.2527 | 0.0054 | 0.1879 |
|  | Leptin | 0.2076 | 0.0229 | 0.2743 |
|  | IL-1β | 0.1857 | 0.0423 | 0.3702 |
|  | Cholesterol intake | -0.2084 | 0.0235 | 0.2743 |
| Acidaminococcaceae | BMI | 0.2592 | 0.0043 | 0.1491 |
|  | IL-1β | 0.2341 | 0.0101 | 0.1764 |
| Verrucomicrobiaceae | BMI | -0.1887 | 0.0390 | 0.1949 |
|  | **Resistin** | **-0.3158** | **0.0004** | **0.0077** |
|  | Leptin | -0.2053 | 0.0245 | 0.1428 |
|  | Insulin | -0.2293 | 0.0118 | 0.0978 |
|  | HOMA-IR | -0.2407 | 0.0086 | 0.0978 |
|  | **IL-1β** | **-0.4353** | **<0.0001** | **<0.0001** |
|  | Subcutaneous fat | -0.2258 | 0.0140 | 0.0978 |
| Lactobacillaceae | Adiponectin | -0.1814 | 0.0473 | 0.2761 |
|  | Resistin | -0.2323 | 0.0107 | 0.1771 |
|  | Leptin | -0.2125 | 0.0198 | 0.1771 |
|  | **IL-1β** | **-0.3559** | **0.0001** | **0.0023** |
|  | Subcutaneous fat | -0.2135 | 0.0202 | 0.1771 |
|  | DEXA-2 | -0.1859 | 0.0429 | 0.2761 |
| Comamonadaceae | WC | -0.2030 | 0.0261 | 0.1617 |
|  | Adiponectin | -0.2332 | 0.0104 | 0.0906 |
|  | Resistin | -0.1907 | 0.0370 | 0.1617 |
|  | Leptin | -0.1980 | 0.0302 | 0.1617 |
|  | **Insulin** | **-0.3083** | **0.0006** | **0.0072** |
|  | **HOMA-IR** | **-0.3190** | **0.0004** | **0.0072** |
|  | **IL-1β** | **-0.4598** | **<0.0001** | **<0.0001** |
|  | Subcutaneous fat | -0.1934 | 0.0359 | 0.1617 |
| Corynebacteriaceae | HDL-C | -0.2389 | 0.0086 | 0.1504 |
|  | ApoA1 | -0.2756 | 0.0023 | 0.0809 |
|  | ApoB/ApoA1 | 0.1896 | 0.0380 | 0.3327 |
|  | Leptin | -0.1979 | 0.0303 | 0.3327 |
| Bifidobacteriaceae | AST | -0.2001 | 0.0284 | 0.3224 |
|  | ALT | -0.1882 | 0.0395 | 0.3224 |
|  | **Resistin** | **0.3054** | **0.0007** | **0.0243** |
|  | Subcutaneous fat | 0.2224 | 0.0155 | 0.2709 |
| Enterococcaceae | HDL-C | -0.2065 | 0.0236 | 0.3036 |
|  | IL-1β | -0.2032 | 0.0260 | 0.3036 |
|  | Subcutaneous fat | 0.2185 | 0.0174 | 0.3036 |
| Ruminococcaceae | sBP | 0.2189 | 0.0163 | 0.2508 |
|  | dBP | 0.1879 | 0.0399 | 0.2508 |
|  | LDL-C | 0.2028 | 0.0263 | 0.2508 |
|  | ApoB/ApoA1 | 0.1798 | 0.0494 | 0.2508 |
|  | **IL-1β** | **0.2946** | **0.0011** | **0.0382** |
| Mitochondria | DEXA-2 | -0.1962 | 0.0325 | 0.7802 |
| Propionibacteriaceae | LDL-C | -0.1886 | 0.0391 | 0.6315 |
| Coriobacteriaceae | Resistin | 0.2369 | 0.0092 | 0.3212 |
| Flavobacteriaceae | WC | -0.2181 | 0.0167 | 0.1462 |
|  | Adiponectin | -0.2955 | 0.0010 | 0.0184 |
|  | Resistin | -0.2036 | 0.0257 | 0.1502 |
|  | Leptin | -0.1840 | 0.0442 | 0.1719 |
|  | Insulin | -0.1841 | 0.0441 | 0.1719 |
|  | HOMA-IR | -0.1945 | 0.0348 | 0.1719 |
|  | **IL-1β** | **-0.4388** | **<0.0001** | **<0.0001** |
|  | **Subcutaneous fat** | **-0.2764** | **0.0024** | **0.0285** |
|  | DEXA-2 | -0.2094 | 0.0223 | 0.1502 |
| Micrococcaceae | Serotonin | -0.2014 | 0.0274 | 0.5896 |
| Christensenellaceae | **ApoA1** | **-0.3147** | **0.0005** | **0.0162** |
|  | ApoB/ApoA1 | 0.1827 | 0.0458 | 0.4003 |
|  | Adiponectin | 0.1958 | 0.0321 | 0.3740 |
|  | IL-1β | 0.2703 | 0.0028 | 0.0496 |
| Erysipelotrichaceae | Leptin | -0.2249 | 0.0135 | 0.3762 |
|  | IL-6 | 0.2004 | 0.0282 | 0.3762 |
|  | DEXA-2 | -0.1965 | 0.0322 | 0.3762 |
| Bacteroidaceae | Leptin | -0.2258 | 0.0132 | 0.4606 |
| Enterobacteriaceae | BMI | -0.1905 | 0.0372 | 0.2236 |
|  | WC | -0.2063 | 0.0238 | 0.2137 |
|  | ALT | -0.1860 | 0.0419 | 0.2236 |
|  | LDL-C | -0.2135 | 0.0192 | 0.2137 |
|  | Adiponectin | -0.1836 | 0.0447 | 0.2236 |
|  | **IL-1β** | **-0.5170** | **<0.0001** | **<0.0001** |
|  | Visceral fat | -0.2071 | 0.0244 | 0.2137 |
| Peptostreptococcaceae | ApoB | 0.1811 | 0.0478 | 0.3478 |
|  | DEXA-2 | -0.2023 | 0.0274 | 0.3478 |
| Deferribacteraceae | Adiponectin | -0.2079 | 0.0227 | 0.1189 |
|  | **Resistin** | **-0.3308** | **0.0002** | **0.0039** |
|  | Leptin | -0.2340 | 0.0101 | 0.1179 |
|  | Insulin | -0.2063 | 0.0238 | 0.1189 |
|  | HOMA-IR | -0.2173 | 0.0181 | 0.1189 |
|  | **IL-1β** | **-0.4185** | **<0.0001** | **0.0001** |
|  | Subcutaneous fat | -0.2123 | 0.0210 | 0.1189 |
| Clostridiaceae 1 | Adiponectin | 0.2221 | 0.0148 | 0.3229 |
|  | Cholesterol intake | -0.2166 | 0.0185 | 0.3229 |
| Moraxellaceae | BMI | -0.1818 | 0.0469 | 0.3540 |
|  | **Adiponectin** | **-0.2815** | **0.0018** | **0.0323** |
|  | **IL-1β** | **-0.5038** | **<0.0001** | **<0.0001** |
| Xanthomonadaceae | BMI | -0.1833 | 0.0451 | 0.1973 |
|  | TG | -0.2619 | 0.0039 | 0.0676 |
|  | TG to HDL ratio | -0.2407 | 0.0096 | 0.0836 |
|  | Insulin | -0.2002 | 0.0283 | 0.1428 |
|  | HOMA-IR | -0.2451 | 0.0075 | 0.0836 |
|  | **IL-1β** | **-0.3524** | **0.0001** | **0.0028** |
|  | Visceral fat | -0.2122 | 0.0211 | 0.1428 |
|  | Saturated fatty acid intake | -0.2016 | 0.0286 | 0.1428 |
| **Genus level** |  |  |  |  |
| *Prevotella 9* | IL-1β | 0.1870 | 0.0408 | 0.6967 |
| [Ruminococcus] torques group | Subcutaneous fat | 0.1946 | 0.0347 | 0.8813 |
| *Haemophilus* | Resistin | -0.2116 | 0.0204 | 0.3672 |
|  | IL-1β | -0.2106 | 0.0210 | 0.3672 |
|  | Subcutaneous fat | -0.1849 | 0.0450 | 0.5253 |
| *Blautia* | IL-1β | -0.2395 | 0.0084 | 0.2946 |
| *Staphylococcus* | Adiponectin | -0.2008 | 0.0279 | 0.4240 |
|  | **IL-1β** | **-0.4679** | **<0.0001** | **<0.0001** |
| *Pseudomonas* | BMI | -0.2536 | 0.0052 | 0.0607 |
|  | Adiponectin | -0.2670 | 0.0032 | 0.0560 |
|  | **IL-1β** | **-0.5087** | **<0.0001** | **<0.0001** |
| *Alistipes* | BMI | 0.1804 | 0.0486 | 0.2127 |
|  | AST | -0.1884 | 0.0393 | 0.2127 |
|  | ApoA1 | -0.2202 | 0.0156 | 0.1763 |
|  | Adiponectin | 0.2700 | 0.0029 | 0.0501 |
|  | **IL-1β** | **0.4179** | **<0.0001** | **0.0001** |
|  | Lipid intake | 0.1895 | 0.0398 | 0.2127 |
|  | Cholesterol intake | 0.2137 | 0.0202 | 0.1763 |
|  | Polyunsaturated fatty acid intake | 0.1824 | 0.0481 | 0.2127 |
| *Dialister* | Resistin | 0.2964 | 0.0010 | 0.0177 |
|  | **IL-1β** | **0.3199** | **0.0004** | **0.0129** |
| *Butyricicoccus* | LDL-C | 0.2088 | 0.0221 | 0.3023 |
|  | ApoB | 0.2033 | 0.0259 | 0.3023 |
|  | IL-1β | 0.2707 | 0.0028 | 0.0977 |
|  | Calcium intake | -0.1891 | 0.0403 | 0.3524 |
| Ruminococcaceae UCG-014 | BMI | -0.2081 | 0.0226 | 0.1975 |
|  | WC | -0.1831 | 0.0454 | 0.2646 |
|  | Resistin | -0.2508 | 0.0057 | 0.0669 |
|  | Leptin | -0.1869 | 0.0410 | 0.2646 |
|  | **IL-1β** | **-0.3706** | **0.0000** | **0.0011** |
|  | Subcutaneous fat | -0.2626 | 0.0041 | 0.0669 |
| *Phascolarctobacterium* | BMI | 0.2592 | 0.0043 | 0.1491 |
|  | IL-1β | 0.2341 | 0.0101 | 0.1764 |
| *Ruminococcus 1* | **Leptin** | **-0.2811** | **0.0019** | **0.0328** |
|  | **DEXA-2** | **-0.2923** | **0.0013** | **0.0328** |
| *Anaerostipes* | DEXA-2 | -0.2178 | 0.0173 | 0.5813 |
|  | Cholesterol intake | 0.1857 | 0.0440 | 0.5813 |
| *Akkermansia* | BMI | -0.1887 | 0.0390 | 0.1949 |
|  | **Resistin** | **-0.3158** | **0.0004** | **0.0077** |
|  | Leptin | -0.2053 | 0.0245 | 0.1428 |
|  | Insulin | -0.2293 | 0.0118 | 0.0978 |
|  | HOMA-IR | -0.2407 | 0.0086 | 0.0978 |
|  | **IL-1β** | **-0.4353** | **<0.0001** | **<0.0001** |
|  | Subcutaneous fat | -0.2258 | 0.0140 | 0.0978 |
| *Lactobacillus* | Adiponectin | -0.1814 | 0.0473 | 0.2761 |
|  | Resistin | -0.2323 | 0.0107 | 0.1771 |
|  | Leptin | -0.2125 | 0.0198 | 0.1771 |
|  | **IL-1β** | **-0.3559** | **0.0001** | **0.0023** |
|  | Subcutaneous fat | -0.2135 | 0.0202 | 0.1771 |
|  | DEXA-2 | -0.1859 | 0.0429 | 0.2761 |
| *Diaphorobacter* | WC | -0.2030 | 0.0261 | 0.1617 |
|  | Adiponectin | -0.2332 | 0.0104 | 0.0906 |
|  | Resistin | -0.1907 | 0.0370 | 0.1617 |
|  | Leptin | -0.1980 | 0.0302 | 0.1617 |
|  | **Insulin** | **-0.3083** | **0.0006** | **0.0072** |
|  | **HOMA-IR** | **-0.3190** | **0.0004** | **0.0072** |
|  | **IL-1β** | **-0.4598** | **<0.0001** | **<0.0001** |
|  | Subcutaneous fat | -0.1934 | 0.0359 | 0.1617 |
| *Corynebacterium 1* | HDL-C | -0.2389 | 0.0086 | 0.1504 |
|  | ApoA1 | -0.2756 | 0.0023 | 0.0809 |
|  | ApoB/ApoA1 | 0.1896 | 0.0380 | 0.3327 |
|  | Leptin | -0.1979 | 0.0303 | 0.3327 |
| *Bifidobacterium* | AST | -0.2001 | 0.0284 | 0.3224 |
|  | ALT | -0.1882 | 0.0395 | 0.3224 |
|  | Resistin | 0.3054 | 0.0007 | 0.0243 |
|  | Subcutaneous fat | 0.2224 | 0.0155 | 0.2709 |
| *Enterococcus* | HDL | -0.2065 | 0.0236 | 0.3036 |
|  | IL-1β | -0.2032 | 0.0260 | 0.3036 |
|  | Subcutaneous fat | 0.2185 | 0.0174 | 0.3036 |
| *Faecalibacterium* | BMI | 0.2455 | 0.0069 | 0.0527 |
|  | WC | 0.2428 | 0.0075 | 0.0527 |
|  | WHR | 0.1899 | 0.0378 | 0.1203 |
|  | sBP | 0.1856 | 0.0424 | 0.1235 |
|  | ALT | 0.2298 | 0.0116 | 0.0578 |
|  | **LDL-C** | **0.2637** | **0.0036** | **0.0422** |
|  | ApoB | 0.2370 | 0.0091 | 0.0533 |
|  | ApoB/ApoA1 | 0.2161 | 0.0178 | 0.0691 |
|  | Serotonin | -0.2123 | 0.0199 | 0.0698 |
|  | **IL-1β** | **0.3666** | **0.0000** | **0.0013** |
|  | Visceral fat | 0.2189 | 0.0172 | 0.0691 |
|  | **Subcutaneous fat** | **0.2731** | **0.0028** | **0.0422** |
| Ruminococcaceae UCG-005 | Adiponectin | 0.2707 | 0.0028 | 0.0488 |
|  | IL-1B | 0.2859 | 0.0015 | 0.0488 |
|  | DEXA-2 | -0.1962 | 0.0325 | 0.7802 |
|  | BMI | -0.2155 | 0.0181 | 0.3161 |
| *Subdoligranulum* | BMI | 0.2070 | 0.0233 | 0.1164 |
|  | WC | 0.1893 | 0.0384 | 0.1680 |
|  | dBP | 0.2071 | 0.0232 | 0.1164 |
|  | Resistin | 0.2116 | 0.0203 | 0.1164 |
|  | **Insulin** | **0.2636** | **0.0036** | **0.0317** |
|  | **HOMA-IR** | **0.2696** | **0.0032** | **0.0317** |
|  | **IL-1β** | **0.4183** | **<0.0001** | **0.0001** |
|  | Subcutaneous fat | 0.2923 | 0.0013 | 0.0231 |
| Lachnospiraceae NK4A136 group | Adiponectin | -0.2198 | 0.0159 | 0.0926 |
|  | **Resistin** | **-0.3447** | **0.0001** | **0.0020** |
|  | Insulin | -0.2228 | 0.0144 | 0.0926 |
|  | HOMA-IR | -0.2274 | 0.0133 | 0.0926 |
|  | **IL-1β** | **-0.4388** | **<0.0001** | **<0.0001** |
|  | Subcutaneous fat | -0.2298 | 0.0123 | 0.0926 |
| *Propionibacterium* | LDL-C | -0.1886 | 0.0391 | 0.6315 |
| Lachnospiraceae NC2004 group | ApoA1 | 0.1811 | 0.0477 | 0.5703 |
|  | Serotonin | 0.1802 | 0.0489 | 0.5703 |
|  | **IL-1β** | **0.3280** | **0.0003** | **0.0089** |
| [Eubacterium] coprostanoligenes group | Resistin | -0.2136 | 0.0192 | 0.5356 |
|  | Leptin | -0.1834 | 0.0449 | 0.5356 |
|  | DEXA-2 | -0.1834 | 0.0459 | 0.5356 |
| *Collinsella* | Resistin | 0.2369 | 0.0092 | 0.3212 |
| *Cloacibacterium* | WC | -0.2181 | 0.0167 | 0.1462 |
|  | **Adiponectin** | **-0.2955** | **0.0010** | **0.0184** |
|  | Resistin | -0.2036 | 0.0257 | 0.1502 |
|  | Leptin | -0.1840 | 0.0442 | 0.1719 |
|  | Insulin | -0.1841 | 0.0441 | 0.1719 |
|  | HOMA-IR | -0.1945 | 0.0348 | 0.1719 |
|  | **IL-1β** | **-0.4388** | **<0.0001** | **<0.0001** |
|  | **Subcutaneous fat** | **-0.2764** | **0.0024** | **0.0285** |
|  | DEXA-2 | -0.2094 | 0.0223 | 0.1502 |
| *Klebsiella* | BMI | -0.2191 | 0.0162 | 0.1136 |
|  | WC | -0.2294 | 0.0117 | 0.1024 |
|  | WHR | -0.2064 | 0.0237 | 0.1383 |
|  | LDL-C | -0.1922 | 0.0354 | 0.1550 |
|  | **Adiponectin** | **-0.2889** | **0.0014** | **0.0240** |
|  | **IL-1β** | **-0.5566** | **<0.0001** | **<0.0001** |
|  | Visceral fat | -0.1958 | 0.0336 | 0.1550 |
|  | Subcutaneous fat | -0.2535 | 0.0056 | 0.0654 |
| *Micrococcus* | Serotonin | -0.2014 | 0.0274 | 0.5896 |
| *Peptoclostridium* | Adiponectin | 0.2103 | 0.0211 | 0.7401 |
| *Ruminiclostridium 6* | BMI | -0.2282 | 0.0122 | 0.0610 |
|  | **WC** | **-0.2478** | **0.0064** | **0.0440** |
|  | **Resistin** | **-0.3316** | **0.0002** | **0.0038** |
|  | **Insulin** | **-0.2428** | **0.0075** | **0.0440** |
|  | **HOMA-IR** | **-0.2559** | **0.0052** | **0.0440** |
|  | **IL-1β** | **-0.4807** | **<0.0001** | **<0.0001** |
|  | **Subcutaneous fat** | **-0.2700** | **0.0031** | **0.0362** |
|  | DEXA-2 | -0.1830 | 0.0463 | 0.2028 |
| *Enterobacter* | WC | -0.2008 | 0.0279 | 0.2394 |
|  | Resistin | -0.1935 | 0.0342 | 0.2394 |
|  | Leptin | -0.1863 | 0.0416 | 0.2426 |
|  | Insulin | -0.2041 | 0.0254 | 0.2394 |
|  | HOMA-IR | -0.2147 | 0.0195 | 0.2394 |
|  | **IL-1β** | **-0.4946** | **<0.0001** | **<0.0001** |
| [Eubacterium] rectale group | Resistin | 0.2789 | 0.0020 | 0.0714 |
|  | Energy intake | -0.2355 | 0.0102 | 0.1656 |
|  | Carbohydrate intake | -0.2252 | 0.0142 | 0.1656 |
| Ruminococcaceae UCG-002 | Adiponectin | 0.2549 | 0.0050 | 0.0869 |
|  | Resistin | 0.2130 | 0.0195 | 0.2276 |
|  | **IL-1β** | **0.3337** | **0.0002** | **0.0068** |
| *Dorea* | Adiponectin | 0.1839 | 0.0444 | 0.5229 |
|  | IL-1β | 0.2390 | 0.0086 | 0.3001 |
| Christensenellaceae R-7 group | **ApoA1** | **-0.3147** | **0.0005** | **0.0162** |
|  | ApoB/ApoA1 | 0.1827 | 0.0458 | 0.4003 |
|  | Adiponectin | 0.1958 | 0.0321 | 0.3740 |
|  | **IL-1β** | **0.2703** | **0.0028** | **0.0496** |
| *Turicibacter* | ApoB/ApoA1 | 0.2018 | 0.0271 | 0.3159 |
|  | **Leptin** | **-0.2956** | **0.0010** | **0.0365** |
|  | DEXA-2 | -0.2606 | 0.0042 | 0.0736 |
| [Ruminococcus] gnavus group | IL-6 | 0.2776 | 0.0021 | 0.0751 |
| [Eubacterium] hallii group | Resistin | 0.2679 | 0.0031 | 0.1084 |
|  | IL-1B | 0.2115 | 0.0204 | 0.3566 |
|  | Subcutaneous fat | 0.1884 | 0.0410 | 0.4464 |
| *Bacteroides* | Leptin | -0.2258 | 0.0132 | 0.4606 |
| *Escherichia-Shigella* | BMI | -0.2684 | 0.0030 | 0.0531 |
|  | WC | -0.2118 | 0.0202 | 0.1771 |
|  | Adiponectin | -0.2388 | 0.0086 | 0.1006 |
|  | **IL-1β** | **-0.5054** | **<0.0001** | **<0.0001** |
|  | Subcutaneous fat | -0.1812 | 0.0496 | 0.2958 |
| *Veillonella* | Fiber intake | -0.1819 | 0.0486 | 0.3443 |
|  | Folate intake | -0.2558 | 0.0052 | 0.1812 |
|  | Cholesterol intake | -0.1994 | 0.0304 | 0.3443 |
| Erysipelotrichaceae UCG-003 | Resistin | 0.1916 | 0.0360 | 0.6951 |
|  | Subcutaneous fat | 0.1840 | 0.0461 | 0.6951 |
| *Fusicatenibacter* | BMI | 0.2141 | 0.0189 | 0.2034 |
|  | ALT | 0.1929 | 0.0348 | 0.2291 |
|  | Resistin | 0.2520 | 0.0055 | 0.0963 |
|  | IL-1β | 0.2527 | 0.0054 | 0.0963 |
|  | Subcutaneous fat | 0.2088 | 0.0232 | 0.2034 |
|  | Protein intake | -0.1886 | 0.0408 | 0.2291 |
| *Roseburia* | Adiponectin | 0.1951 | 0.0327 | 0.5985 |
|  | Resistin | 0.1807 | 0.0483 | 0.5985 |
| *Intestinibacter* | DEXA-2 | -0.2133 | 0.0198 | 0.4711 |
|  | Calcium intake | -0.1899 | 0.0394 | 0.4711 |
| *Ruminiclostridium 9* | Resistin | 0.2441 | 0.0072 | 0.1261 |
|  | **IL-1β** | **0.3464** | **0.0001** | **0.0037** |
| Lachnospiraceae  UCG-008 | **Resistin** | **0.3633** | **<0.0001** | **0.0016** |
| *Mucispirillum* | Adiponectin | -0.2079 | 0.0227 | 0.1189 |
|  | **Resistin** | **-0.3308** | **0.0002** | **0.0039** |
|  | Leptin | -0.2340 | 0.0101 | 0.1179 |
|  | Insulin | -0.2063 | 0.0238 | 0.1189 |
|  | HOMA-IR | -0.2173 | 0.0181 | 0.1189 |
|  | **IL-1β** | **-0.4185** | **<0.0001** | **0.0001** |
|  | Subcutaneous fat | -0.2123 | 0.0210 | 0.1189 |
| *Lachnoclostridium* | WC | 0.2033 | 0.0260 | 0.2617 |
|  | Insulin | 0.1946 | 0.0331 | 0.2617 |
|  | HOMA-IR | 0.2025 | 0.0279 | 0.2617 |
|  | **IL-1β** | **0.3042** | **0.0007** | **0.0256** |
|  | Subcutaneous fat | 0.1917 | 0.0376 | 0.2617 |
|  | Saturated fatty acid intake | 0.1850 | 0.0449 | 0.2617 |
| Ruminococcaceae NK4A214 group | **IL-1β** | **0.3817** | **<0.0001** | **0.0006** |
|  | Lipid | 0.2418 | 0.0083 | 0.1459 |
|  | Polyunsaturated fatty acid intake | 0.1985 | 0.0311 | 0.3632 |
| *Clostridium*  *sensu stricto 1* | Adiponectin | 0.2221 | 0.0148 | 0.3229 |
|  | Cholesterol intake | -0.2166 | 0.0185 | 0.3229 |
| *Acinetobacter* | BMI | -0.1818 | 0.0469 | 0.3540 |
|  | Adiponectin | -0.2815 | 0.0018 | 0.0323 |
|  | **IL-1β** | **-0.5038** | **<0.0001** | **<0.0001** |
| *Stenotrophomonas* | BMI | -0.1833 | 0.0451 | 0.1973 |
|  | TG | -0.2619 | 0.0039 | 0.0676 |
|  | TG/HDL-C ratio | -0.2407 | 0.0096 | 0.0836 |
|  | Insulin | -0.2002 | 0.0283 | 0.1428 |
|  | HOMA-IR | -0.2451 | 0.0075 | 0.0836 |
|  | **IL-1β** | **-0.3524** | **0.0001** | **0.0028** |
|  | Visceral fat | -0.2122 | 0.0211 | 0.1428 |
|  | Saturated fatty acid intake | -0.2016 | 0.0286 | 0.1428 |

Correlation between different clinical parameters and bacterial abundance was analyzed by Spearman’s rank correlation analysis. Correlations showing *p*-value < 0.05 is summarized in the supplementary table. Statistically significant correlation with FDR q*-*value < 0.05 are indicated by bold style.

BMI, body mass index; sBP, systolic blood pressure; dBP diastolic blood pressure; LDL-C, low-density lipoprotein cholesterol; HDL-C, high-density lipoprotein cholesterol; TG, triglyceride, AST, aspartate aminotransferase; ALT, alanine aminotransferase; ApoA1, apolipoprotein A1; ApoB, apolipoprotein B; HOMA-IR, Homeostatic Model Assessment for Insulin Resistance; HOMA-β, Homeostasis model assessment of β-cell function; IL-6, interleukin-6; IL-1β, interleukin-Iβ; FDR, false discovery rate calculated by Benjamini-Hochberg method; IQR, interquartile range.

**Supplementary table 2**. Taxa showing a significant different abundance between enterotypes in the EV-derived microbiome.

| Taxon | t-statistic | *p-*value | FDR q-value |
| --- | --- | --- | --- |
| **Enterotype I > Enterotype II** |  |  |  |
| Phylum Bacteroidetes | -7.7807 | 0.0001 | 0.0002 |
| Family Prevotellaceae | -8.4155 | 0.0001 | 0.0003 |
| Genus *Prevotella* | -8.4155 | 0.0001 | 0.0004 |
| **Enterotype II > Enterotype I** |  |  |  |
| Family Lanchnospiraceae | 5.6724 | 0.0001 | 0.0003 |
| Genus *Anaerostipes* | 4.9182 | 0.0001 | 0.0004 |
| Genus *Lachnospiraceae NK4A136 group* | 3.7971 | 0.0002 | 0.0006 |
| Genus *Blautia* | 3.3138 | 0.0002 | 0.0006 |
| Family Lactobacillaceae | 3.9427 | 0.0001 | 0.0003 |
| Genus *Lactobacillus* | 3.9427 | 0.0001 | 0.0004 |
| Family Peptostreptococcaceae |  |  |  |
| Genus *Intestinibacter* | 4.0476 | 0.0001 | 0.0004 |
| Family Ruminococcaceae |  |  |  |
| Genus *Ruminococcaceae UCG-013* | 5.2065 | 0.0001 | 0.0004 |
| Genus *Ruminococcaceae UCG-014* | 4.8483 | 0.0001 | 0.0004 |
| Genus *Ruminococcus 2* | 4.0155 | 0.0003 | 0.0009 |
| Genus *Ruminiclostridium 6* | 3.2148 | 0.0009 | 0.0024 |
| Family Staphylococcaceae | 3.7273 | 0.0002 | 0.0005 |
| Genus *Staphylococcus* | 3.7273 | 0.0002 | 0.0006 |
| Family Streptococcaceae | 4.2106 | 0.0001 | 0.0003 |
| Genus *Streptococcus* | 4.2106 | 0.0001 | 0.0004 |
| Phylum Bacteroidetes |  |  |  |
| Family Bacteroidaceae | 4.8571 | 0.0001 | 0.0003 |
| Genus *Bacteroides* | 4.8571 | 0.0001 | 0.0004 |
| Family Flavobacteriaceae | 2.9498 | 0.0018 | 0.0040 |
| Family Porphyromonadaceae | 3.0633 | 0.0014 | 0.0034 |
| Genus *Parabacteroides* | 3.0633 | 0.0014 | 0.0034 |
| Phylum Actinobacteria | 2.3645 | 0.0174 | 0.0232 |
| Phylum Deferribacteres | 4.2514 | 0.0001 | 0.0002 |
| Family Deferribacteraceae | 4.2514 | 0.0001 | 0.0003 |
| Genus *Mucispirillum* | 4.2514 | 0.0001 | 0.0004 |
| Phylum Proteobacteria | 4.2747 | 0.0001 | 0.0002 |
| Family Comamonadaceae | 2.9119 | 0.0027 | 0.0052 |
| Family Enterobacteriaceae | 4.2863 | 0.0001 | 0.0003 |
| Genus *Enterobacter* | 4.5921 | 0.0001 | 0.0004 |
| Genus *Klebsiella* | 3.8088 | 0.0001 | 0.0004 |
| Genus *Escherichia-Shigella* | 3.4477 | 0.0008 | 0.0023 |
| Family Moraxellaceae | 4.1058 | 0.0001 | 0.0003 |
| Genus *Actinobacter* | 4.1058 | 0.0001 | 0.0004 |
| Family Pseudomonadaceae | 2.9410 | 0.0024 | 0.0050 |
| Genus *Pseudomonas* | 4.0650 | 0.0001 | 0.0004 |
| Phylum Verrucomicrobia | 4.6358 | 0.0001 | 0.0002 |
| Family Verrucomicrobiaceae | 4.6358 | 0.0001 | 0.0003 |
| Genus *Akkermansia* | 4.6358 | 0.0001 | 0.0004 |
| Phylum Firmicutes | 4.1407 | 0.0001 | 0.0002 |
| Family Bacilli | 5.3113 | 0.0001 | 0.0004 |
| Family Erysipelotrichia | 3.5875 | 0.0002 | 0.0005 |
| Family Clostridia | 3.6224 | 0.0003 | 0.0006 |

Differences in microbial abundance were calculated by Wilcoxon rank-sum test, and adjusted *p*-value, FDR; false discovery rate calculated by Benjamini-Hochberg method.

**Supplementary Table 3.** Characteristics of the male and female subjects according to the two enterotypes

| Variables | Male ( n = 21) | | | | Female (n = 99) | | | |
| --- | --- | --- | --- | --- | --- | --- | --- | --- |
|  | Enterotype 1  (n=5) | Enterotype 2  (n=16) | *p*-value | FDR q | Enterotype 1  (n=29) | Enterotype 2  (n=70) | *p*-value | FDR q |
| Age (years) | 38.8 ± 7.8 | 36.3 ± 8.6 | 0.563 | 0.563 | 46.8 ± 8.9 | 44.9 ± 8.1 | 0.203 | 0.609 |
| Smoker, n (%) | 3 (60.0) | 3 (18.8) | 0.115 | 0.345 | 0 (0.0) | 1(1.4) | 0.518 | 0.777 |
| Consumes alcohol^††^,  n (%) | 5 (100.0) | 12 (75.0) | 0.532 | 0.563 | 7 (24.1) | 18 (25.7) | 0.896 | 0.896 |
| BMI (kg/m^2^) | 32.2 ± 1.44 | 28.43 ± 2.2 | **0.002** | **0.010** | 27.8 ± 2.4 | 27.3 ± 2.2 | 0.352 | 0.587 |
| Waist circum. (cm) | 106.9 ± 3.1 | 100.2 ± 1.3 | **0.028** | 0.070 | 94.7 ± 6.9 | 93.3 ± 5.9 | 0.315 | 0.587 |
| Waist/hip circum. (cm) | 0.96 ± 0.04 | 0.94.0 ± 0.03 | 0.278 | 0.463 | 0.92 ± 0.05 | 0.92 ± 0.05 | 0.589 | 0.736 |
| sBP (mmHg) | 137.6 ± 6.5 | 132.7 ± 3.1 | 0.462 | 0.578 | 124.8 ± 10.6 | 124.0 ± 13.4 | 0.767 | 0.767 |
| dBP (mmHg) | 84.8 ± 3.5 | 84.1 ± 2.2 | 0.871 | 0.871 | 73.6 ± 8.1 | 76.3 ± 9.6 | 0.179 | 0.587 |
| Triglyceride (mg/dl) | 180.2 ± 98.0 | 146.6 ± 79.3 | 0.443 | 0.591 | 125.3 ± 61.6 | 113.7 ± 68.2 | 0.430 | 0.860 |
| LDL-C (mg/dl) | 126.2 ± 8.0 | 128.9 ± 5.3 | 0.797 | 0.797 | 116.4 ± 30.1 | 116.4 ± 5.3 | 0.998 | 0.998 |
| HDL-C (mg/dl) | 42.6 ± 6.0 | 52.1 ± 7.9 | **0.024** | 0.096 | 56.2 ± 13.4 | 57.2 ± 10.7 | 0.700 | 0.933 |
| TG/HDL-C | 4.5 ± 1.2 | 2.9 ± 0.3 | 0.087 | 0.174 | 2.5 ± 1.8 | 2.1 ± 1.3 | 0.172 | 0.688 |
| AST (IU/ml) | 37.2 ± 7.3 | 30.4 ± 2.9 | 0.314 | 0.314 | 24.4 ± 9.5 | 23.2 ± 7.5 | 0.486 | 0.486 |
| ALT (IU/ml) | 61.4 ± 10.3 | 45.6 ± 5.7 | 0.192 | 0.314 | 26.1 ± 14.5 | 20.9 ± 12.2 | 0.073 | 0.146 |
| Apo A1^†^ (mg/dl) | 139.5 (128.0 – 154.0) | 143.0 (133.0 – 158.0) | 0.090 | 0.396 | 143 (130 – 156) | 145 (134 – 160) | 0.645 | 0.941 |
| Apo B^†^ (mg/dl) | 97.0 (91.0 – 112.0) | 99.0 (85.0 – 111.0) | **0.027** | 0.324 | 97 (87 – 112) | 96 (82 – 110) | 0.785 | 0.941 |
| ApoB/A1 ratio^†^ | 0.7 (0.6 – 0.9) | 0.7 (0.6 – 0.8) | 0.186 | 0.425 | 0.68 (0.56 – 0.88) | 0.66 (0.52 – 0.79) | 0.615 | 0.941 |
| Adiponectin^†^ (μg/dl) | 6.7 (4.0 – 8.3) | 6.5 (4.0 – 8.2) | 0.231 | 0.425 | 7.0 (4.9 – 9.2) | 6.9 (4 – 8.5) | 0.724 | 0.941 |
| Resistin^†^ (ng/dl) | 6.2 (4.3 – 9.3) | 5.2 (3.8 – 7.1) | 0.364 | 0.485 | 5.9 (4.3 – 9.3) | 5.3 (3.8 – 7.2) | 0.159 | 0.941 |
| Leptin^†^ (ng/dl) | 31.8 (22.7 – 40.2) | 28.6 (22.5 – 40.1) | 0.099 | 0.396 | 32.0 (24.1 – 40.6) | 31.7 (24.6 – 42.2) | 0.863 | 0.941 |
| Serotonin^†^ (ng/dl) | 123.2 (96.9 – 176.1) | 125.5 (92.4 – 172.6) | 0.804 | 0.836 | 122.9 (96.9 – 180.2) | 125.0 (91 – 177.3) | 1.000 | 1.000 |
| Fasting blood sugar  (mg/dl) | 105 (100 – 106) | 105 (93.5 – 113.5) | 0.836 | 0.836 | 97 (90 – 103) | 97.5 (91 – 103) | 0.832 | 0.941 |
| Fasting insulin^†^ (μU/ml) | 7.4 (4.0 – 10.4) | 6.0 (4.3 – 9.3) | 0.302 | 0.453 | 5.8 (4.0 – 9.8) | 5.7 (4.3 – 9.0) | 0.756 | 0.941 |
| HbA1c (%) | 5.7 (5.5 – 5.9) | 5.6 (5.3 – 5.9) | 0.535 | 0.642 | 5.6 (5.4 – 5.9) | 5.5 (5.3 – 5.7) | 0.077 | 0.924 |
| HOMA-IR^†^ | 2.8 (2.7 – 3.7) | 2.1 (1.1 – 3.2) | 0.248 | 0.425 | 1.4 (0.9 – 2.3) | 1.4 (0.9 – 2.3) | 0.720 | 0.941 |
| HOMA-β^†^ | 108 (72.6 – 117.2) | 64.3 (27.3 – 108.1) | 0.160 | 0.425 | 68.7 (44.6 – 106.7) | 62.1 (43.5 – 97.1) | 0.670 | 0.941 |
| IL-6^†^ (pg/dl) | 2.2 (1.7 – 2.2) | 1.8 (1.1 – 2.2) | 0.302 | 0.302 | 1.5 (0.9 – 2.7) | 1.5 (1.1 – 2.8) | 0.405 | 0.405 |
| IL-1β^†^ (pg/dl) | 5.8 (5.5 – 5.9) | 4.7 (1.8 – 5.5) | 0.090 | 0.180 | 2.3 (1.2 – 5.7) | 1.6 (1.0 – 3.1) | 0.050 | 0.100 |
| Visceral fat (cm^2^) | 174.0 ± 26.6 | 156.5 ±10.6 | 0.336 | 0.549 | 115.4 ± 37.8 | 105.6 ± 30.1 | 0.182 | 0.506, |
| Subcutaneous fat (cm^2^) | 337.0 ± 25.4 | 257.6 ± 18.4 | **0.040** | 0.114 | 285.0 ± 82.6 | 271.9 ± 68.7 | 0.422 | 0.506 |
| Visceral /total body fat | 33.7 ± 3.6 | 37.2 ± 2.4 | 0.475 | 0.570 | 29.0 ± 8.3 | 28.7 ± 7.8 | 0.853 | 0.853 |
| Total fat mass (kg) | 34.9 ± 2.3 | 29.1 ± 1.4 | 0.057 | 0.114 | 29.3 ± 5.8 | 27.9 ± 4.9 | 0.225 | 0.506 |
| Body fat percentage (%) | 36.7 ± 0.6 | 32.9 ± 0.9 | **0.042** | 0.114 | 42.5 ± 3.2 | 41.8 ± 3.2 | 0.352 | 0.506 |
| Lean body mass (kg) | 57.8 ± 2.7 | 56.2 ± 1.5 | 0.596 | 0.596 | 37.3 ± 4.5 | 36.5 ± 4.0 | 0.370 | 0.506 |

Categorical variables were expressed as number of subjects (n), (%), and compared using χ^2^-test. Continuous variables are expressed as mean ± standard deviation, and compared using Student’s t-test, unless otherwise noted.

†Phenotypes not following normal distribution were compared using non-parametric analysis: Wilcoxon rank-sum test. These variables are expressed as median (IQR).

^††^ Alcohol consumption includes both current and past alcohol consumption.

BMI, body mass index; sBP, systolic blood pressure; dBP diastolic blood pressure; LDL-C, low-density lipoprotein cholesterol; HDL-C, high-density lipoprotein cholesterol; TG, triglyceride, AST, aspartate aminotransferase; ALT, alanine aminotransferase; ApoA1, apolipoprotein A1; ApoB, apolipoprotein B; HOMA-IR, Homeostatic Model Assessment for Insulin Resistance; HOMA-β, Homeostasis model assessment of β-cell function; IL-6, interleukin-6; IL-1β, interleukin-Iβ; FDR, false discovery rate calculated by Benjamini-Hochberg method; IQR, interquartile range.

Bold style indicates statistical significance.
