# Supplementary material for: Gut microbiome signature of metabolically healthy obese individuals according to anthropometric, metabolic and inflammatory parameters
Source: Sci Rep. 2024 Feb 11;14:3449. doi: 10.1038/s41598-024-53837-z (PMC10859373; doi:10.1038/s41598-024-53837-z)
Supplement: Supplementary file 2 — Supplementary Figures. [file 41598_2024_53837_MOESM2_ESM.docx]

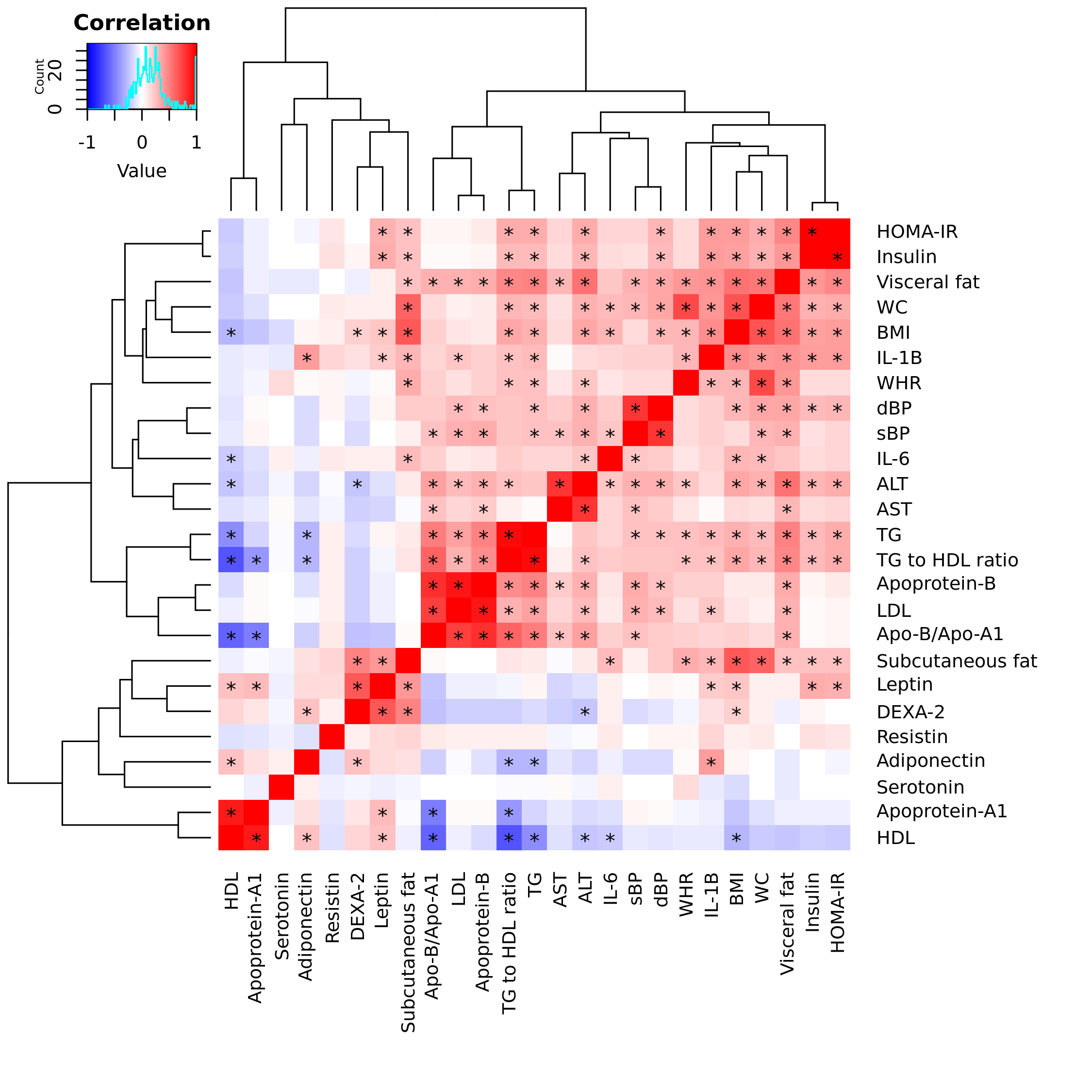


**Supplementary Figure 1. Correlation between different clinical parameters.** Correlation between different clinical parameters were analyzed by Spearman’s correlation analysis. Statistically significant correlation with FDR-q value < 0.05 are indicated by asterisks. Dendrograms on X, Y axis were generated using complete-linkage hierarchical clustering. WC, waist circumference; BMI, body mass index; IL-1B, interleukin-1β; WHR, waist-hip ratio; dBP, diastolic blood pressure; sBP, systolic blood pressure; ALT, alanine aminotransferase; AST, aspartate aminotransferase; TG, triglyceride; HDL, high density lipoprotein; LDL, low density lipoprotein; IL-6, interleukin-6.


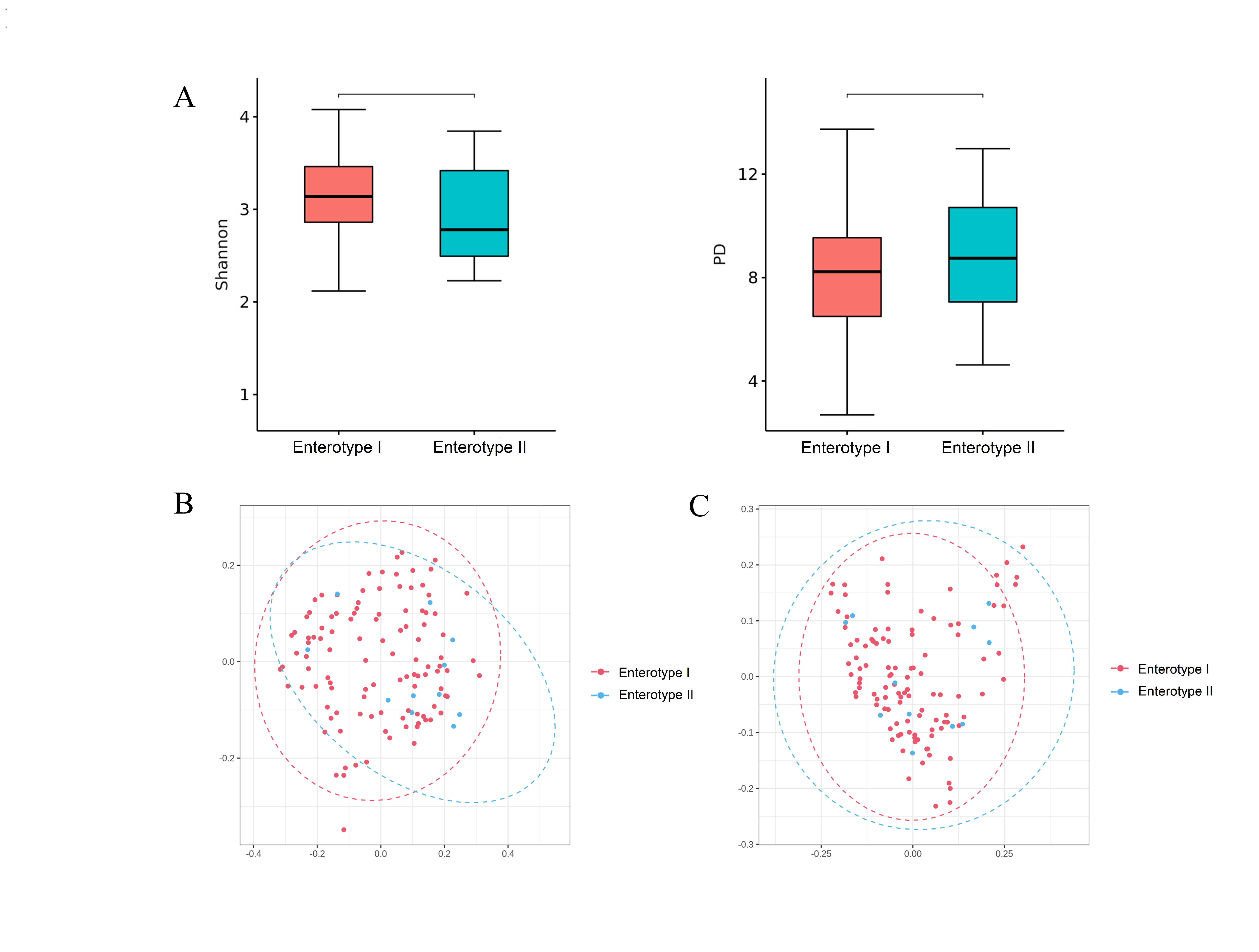


**Supplementary Figure 2. Bacterial cell-originating microbiota diversity (A) and PCoA plots (B, C)** A. Enterotype groups by bacterial cell-originating microbiota did not show significant difference in Shannon index and Phylogenetic diversity (*p* > 0.05), B. Composition of bacterial cell-originating microbiota by Unweighted Unifrac distance (PERMANOVA *p* > 0.05), C. Composition of bacterial cell-originating microbiota by Weighted Unifrac distance (PERMANOVA *p* > 0.05)

**
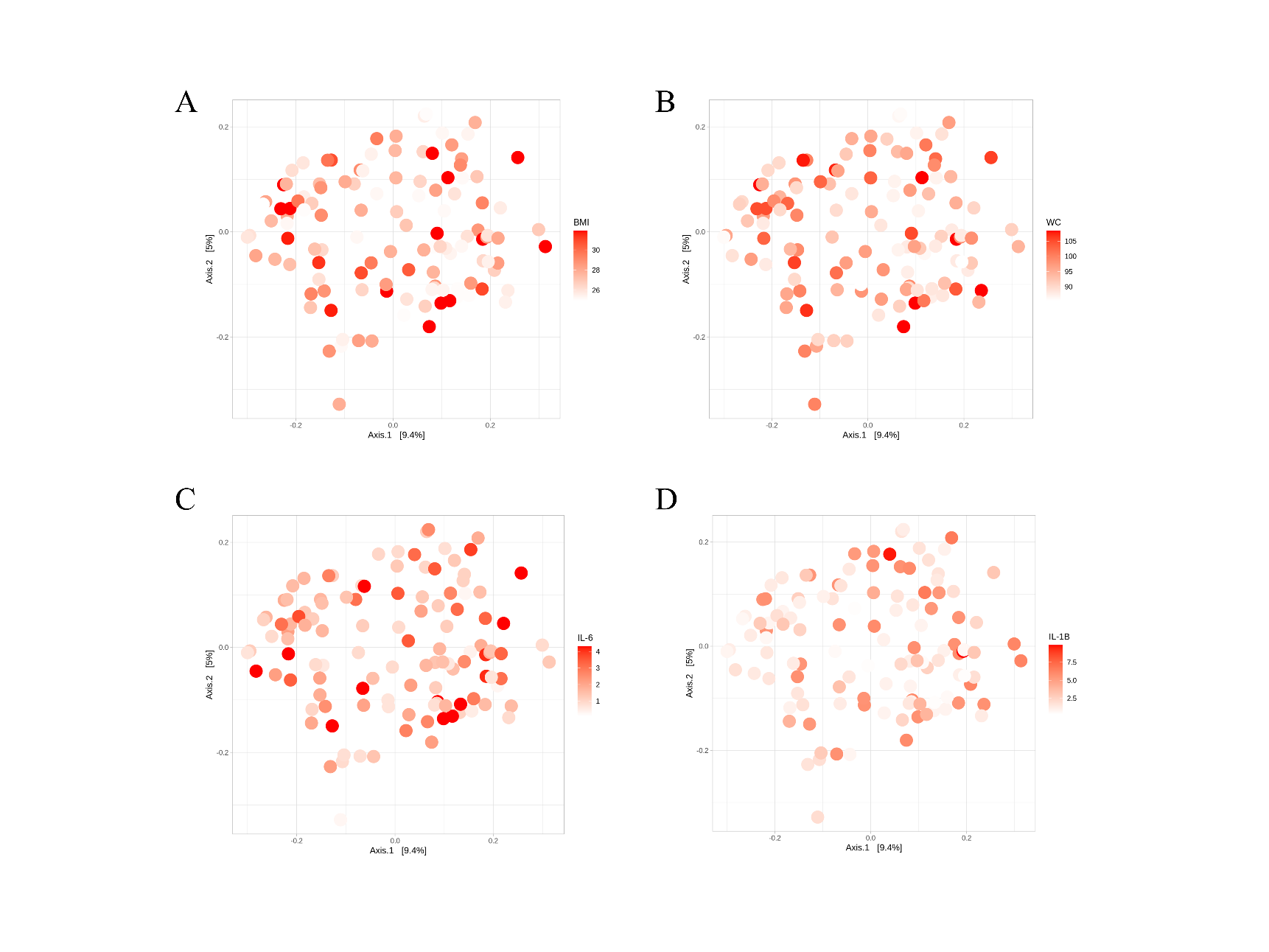
**

**Supplementary Figure S3. Distribution of clinical parameters and inflammatory markers by bacterial cell-derived microbiota composition** A. Weighted UniFrac distance matrix showing distribution of body mass index. B. Weighted UniFrac distance matrix showing distribution of waist circumference. C. Weighted UniFrac distance matrix showing distribution of interleukin-6 D. Weighted UniFrac distance matrix showing distribution of interleukin-1β.
